# Geographic and intra‐racial disparities in early‐onset colorectal cancer in the SEER 18 registries of the United States

**DOI:** 10.1002/cam4.3488

**Published:** 2020-10-22

**Authors:** Wesal H. Abualkhair, Meijiao Zhou, Carolina O. Ochoa, Leonel Lacayo, Caitlin Murphy, Xiao‐Cheng Wu, Jordan J. Karlitz

**Affiliations:** ^1^ Master of Science in Clinical Research School of Medicine Department of Medicine Tulane University New Orleans LA USA; ^2^ Louisiana Tumor Registry and Department of Epidemiology Louisiana State University Health Sciences Center New Orleans LA USA; ^3^ Louisiana State University ‐ Shreveport Shreveport LA USA; ^4^ Gastroenterologist Southeast Louisiana Veterans Health Care Systems New Orleans LA USA; ^5^ Department of Population and Data Sciences University of Texas Southwestern Medical Center Dallas TX USA; ^6^ Public Health and Director of Louisiana Tumor Registry Department of Epidemiology Louisiana State University Health Sciences Center New Orleans LA USA; ^7^ Southeast Louisiana Veterans Health Care System, New Orleans and Division of Gastroenterology, Department of Medicine, Tulane University School of Medicine New Orleans LA USA

**Keywords:** cancer prevention, colorectal cancer, early‐onset, end results (SEER) program, epidemiology, geographic disparities, racial disparities, surveillance

## Abstract

**Background:**

Although early‐onset colorectal cancer (EOCRC) incidence rates (IRs) are increasing, geographic and intra‐racial IR disparities are not well defined.

**Methods:**

2000‐2015 Surveillance, Epidemiology, and End Results (SEER) program CRC IR Analysis (170,434 cases) was performed from ages 30 to 60 in four US regions, 18 individual registries, metropolitan and nonmetropolitan locations and stratified by race. Analyses were conducted in 1‐year and 5‐year age increments.

**Results:**

Wide US regional EOCRC IR variations exist: For example, age 45 IRs in the south are 26.8/100,000, 36.0% higher than the West, 19.7/100,000 (*p* < 0.0001). Disparities magnify between individual registries: EOCRC IRs in highest risk registries were 177‐348% (Alaska Natives), 75‐200% (Hawaii), 76‐128% (Louisiana), and 61‐125% (Kentucky) higher than lowest risk registries depending on age. EOCRC IRs are 18.2%‐25.6% higher in nonmetropolitan versus metropolitan settings. Wide geographic intra‐racial disparities exist. Within the White population, the greatest IR difference (78.8%) was between Kentucky (5.9/100,000) and Los Angeles (3.3/100,000) in 30‐ to 34‐year‐olds (*p* < .0001). Within the Black population, the greatest difference (136.2%) was between rural Georgia (30.7/100,000) and California excluding San Francisco‐Oakland/San Jose‐Monterey/Los Angeles (13/100,000) in 40‐ to 44‐year‐olds (*p* = 0003).

**Conclusion:**

Marked geographic EOCRC disparities exist with disproportionately high IRs in Alaska Natives, Hawaii, and southern registries. Geographic intra‐racial disparities are present within White and Black populations. In Blacks, there are disproportionately high EOCRC IRs in rural Georgia. Although vigilance is required in all populations, attention must be paid to these higher risk populations. Potential interventions include assuring early investigation of symptoms, targeting modifiable risk factors and utilizing earlier age 45 screening options supported by some guidelines.

## Graphical abstract

This analysis of SEER 18 early‐onset colorectal cancer data reveals marked disparities by geography and within racial groups (intra‐racial) across the United States. This data can be used to target public health interventions and guide future studies on etiology.

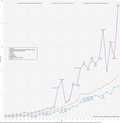

## 1 INTRODUCTION

EOCRC (generally defined as those under age 50) incidence rates (IRs) have been rising, prompting the American Cancer Society (ACS), in contrast to other groups, to recommend average‐risk screening in all patients at age 45.^1^, ^2^, ^3^, ^4^, ^5^, ^6^ As the EOCRC population is heterogeneous, it is important to target higher risk subpopulations for interventions aimed at prevention and early detection. Important subgroups already known to be at higher risk for EOCRC include those with a family history of CRC or other malignancies, which may require screening at earlier ages.^7^ Race is also a risk factor with Blacks having higher EOCRC rates.^8^ This prompted the United States Multi‐Society Task Force on Colorectal Cancer (USMSTF) to recommend average‐risk screening at age 45 in Blacks, although this is not supported by all guidelines.^6^

The relationship between geography and EOCRC IRs has not been well defined. Assessing geographic incidence disparities may identify large communities, potentially in underserved rural or urban areas, that could derive benefit from targeted population‐based efforts aimed at both prevention, through risk factor modification and earlier screening, and cancer detection at earlier stages. Rural communities may have disproportionately low CRC‐screening rates compared to other areas.^9^ Geography in this context can include comparisons of US regions, states, registries, and metropolitan versus nonmetropolitan populations. Identification of higher risk EOCRC subpopulations may also guide future studies to provide insight on risk factors that may contribute to high EOCRC IRs in certain areas.

Furthermore, the relationship between race, geography, and EOCRC incidence has not been well studied. Epidemiological analysis by race can assess whether any overall geographic EOCRC incidence variations are driven by differences between racial subgroups and allow more precise targeted interventions. Assessment of disparities within racial groups (intra‐racial disparities) is important because there may be subpopulations of higher risk patients within an overall racial group that could be potentially overlooked. For example, in Louisiana, although CRC incidence in Whites is higher compared to most US states, a subpopulation in the southern Acadian region was recently demonstrated to have disproportionately higher incidence compared to Louisiana as a whole, including under age 50.^10^ In racial groups already at higher risk for EOCRC, for example the Black population, there may be subpopulations with disproportionately high EOCRC rates, compounding disparities.

Hence, our study goals were twofold. First, we utilized 2000‐2015 US Surveillance, Epidemiology, and End Results (SEER) 18 data to assess EOCRC IRs stratified by US region, individual cancer registry and metropolitan versus nonmetropolitan location. Second, we sub‐stratified EOCRC incidence by race and assessed differences between US regions, individual SEER registries and metropolitan/nonmetropolitan settings.

The SEER 18 database was utilized specifically due to its focus on unique and potentially underserved populations, including Alaska Natives and nonmetropolitan populations (i.e., Georgia). Furthermore, it has publicly available data allowing analyses to be performed in 1‐year age increments as opposed to age blocks/ranges (i.e., age 40‐49). This has the advantage of helping ensure potentially high‐risk regions and registries at a given age are not masked due to incorporation within a larger patient age analysis block.

## 2 METHODS

We analyzed 2000‐2015 cancer incidence data from the 18 population‐based SEER registries.^11^ Data from 2000 onwards were utilized as this encompasses periods of increasing EOCRC rates and when screening became commonplace.^12^ Eligible cases included adenocarcinomas between ages 30 and 60 (Supplmental Info File).

We included the age 50‐60 group (as opposed to just including those under age 50), so we would be able to put into better context the findings in those under age 50. Furthermore, we wanted to include patients in their 50 s because screening rates are not optimized and in 50‐ to 54‐year‐olds in particular, colorectal cancer IRs have been found to be increasing in a similar fashion to younger patients. Rates were not examined under age 30 due to low case counts. Ages over 60 were not assessed as goals are to understand EOCRC. Annual average, age‐adjusted CRC IRs per 100,000 (2000 US standard population) were calculated using SEER*Stat software version 8.3.5 (Bethesda, MD). The *p*‐values less than 0.05 were considered statistically significant. 95% confidence interval calculations were referred to Tiwari et al. 2006.^13^

US regions were grouped according to the US Census Bureau (Southern, Midwestern, Northeastern, and Western).^14^ Metropolitan versus nonmetropolitan categorization is standardly defined as residential address at the time of diagnosis.^15^ Rural‐Urban Continuum Codes (RUCCs) for 2003 developed by the United States Department of Agriculture (USDA) were used to define the metropolitan and nonmetropolitan counties. The metropolitan counties were defined by the population size of their metro area. Nonmetropolitan counties were defined by the degree of urbanization and adjacency to a metro area or areas. The RUCC definitions used in SEER*Stat for nonmetropolitan counties are RUCCs of four to nine, and for metropolitan counties definitions used are RUCCs of one to three.^15^, ^16^, ^17^

Incidence rates by 1‐year age increments were utilized to compare overall geographic regions and individual registries. Due to lower case counts, analysis focused on 5‐year age group blocks for racial sub‐stratification of regions, individual registries, and metropolitan/nonmetropolitan comparisons. However, detailed 1‐year age increment data by race within each registry are also provided in supplemental spreadsheets (see results below). Age‐range cells with case counts less than 15 were excluded.

Race stratification was performed between White and Black populations. Additional race stratification to assess other patient groups (Asian American, Native American, Hispanics alone etc.) was not assessed due to lower case counts (with the exception of the SEER Alaska registry which consists entirely of Alaska Natives). Intra‐racial geographic disparities within both White and Black populations were assessed by calculating the percentage difference between the highest and lowest IRs by region, individual registry, and metropolitan/nonmetropolitan settings.

## 3 RESULTS

There were 170,434 CRC cases overall. The combined number for Whites and Blacks specifically was 152,982.

**Figure 1:**
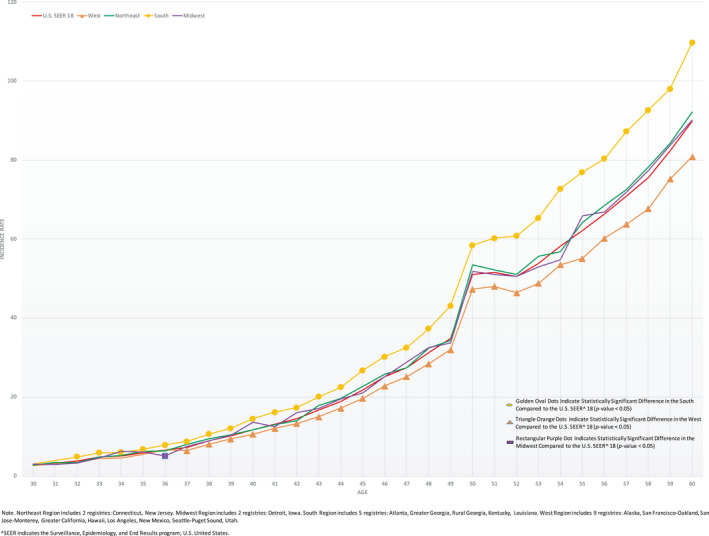
2000‐2015 Colorectal Cancer Incidence Rates per 100,000 in 1‐Year Age Increments for United States Regions (West, South, Northeast, Midwest) Compared to US SEER^ 18, Age 30‐60. ^SEER indicates the Surveillance, Epidemiology, and End Results program; US denotes United States. *Northeast Region includes two registries: Connecticut and New Jersey. Midwest Region includes two registries: Detroit and Iowa. South Region includes five registries: Atlanta, Greater Georgia, Rural Georgia, Kentucky, and Louisiana. West Region includes nine registries: Alaska, San Francisco‐Oakland, San Jose‐Monterey, Greater California, Hawaii, Los Angeles, New Mexico, Seattle‐Puget Sound, and Utah.

**Figure 2:**
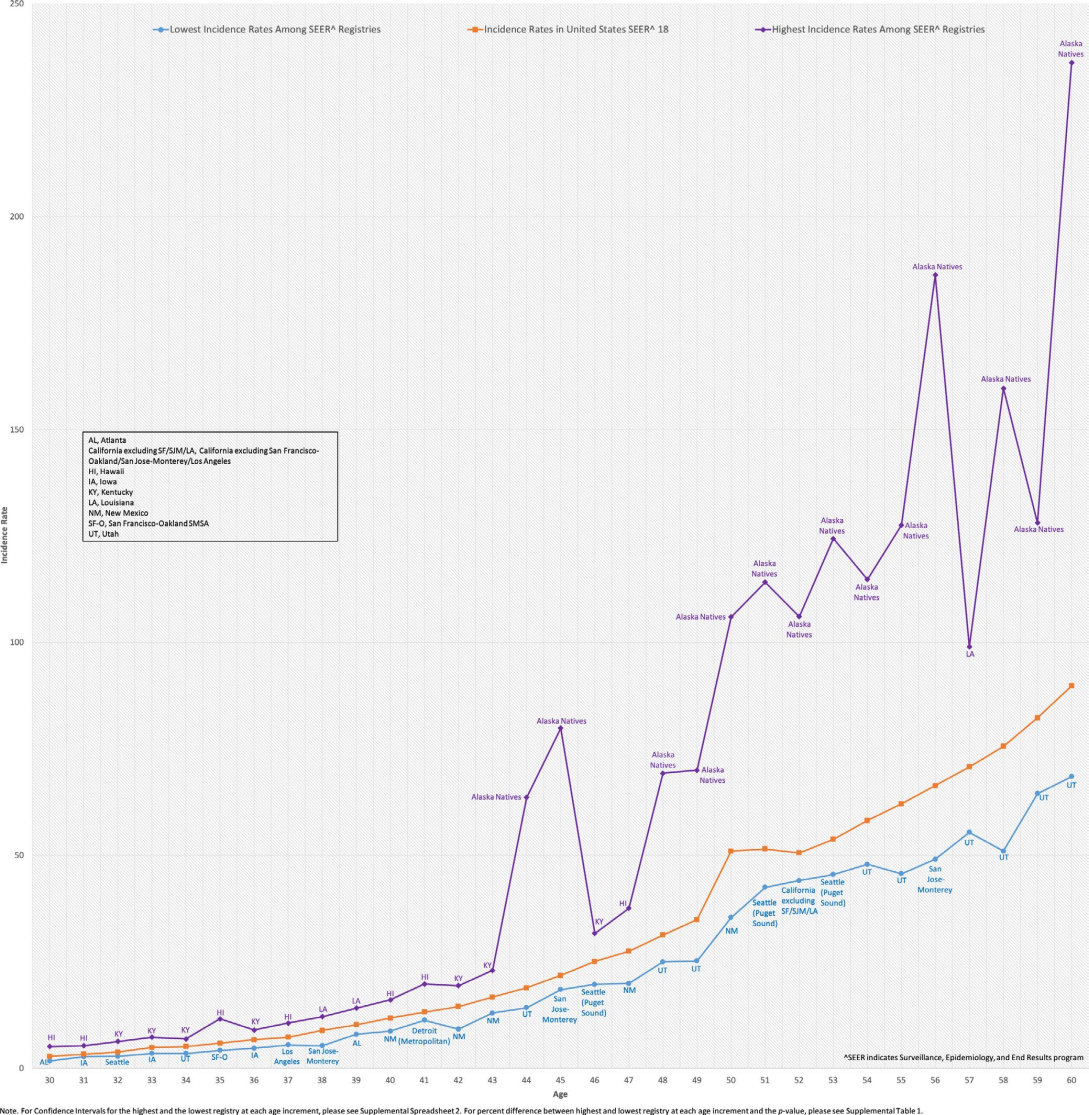
2000‐2015 Colorectal Cancer Incidence Rates per 100,000 in 1‐Year Age Increments for Registries with Lowest Incidence Rates Versus Highest Incidence Rates, Age 30‐60. ^SEER indicates Surveillance, Epidemiology, and End Results program. AL, Atlanta; California excluding SF/SJM/LA, California excluding San Francisco‐Oakland/San Jose‐Monterey/Los Angeles; HI, Hawaii; IA, Iowa; KY, Kentucky; LA, Louisiana; NM, New Mexico; SF‐O, San Francisco‐Oakland SMSA; UT, Utah. Note. For Confidence Intervals for the highest and the lowest registry at each age increment, please see Spreadsheet S2. For percent difference between highest and lowest registry at each age increment and the *p*‐value, please see Table S1.

Wide‐regional variations in EOCRC incidence exist (Figure 1, Spreadsheet S1). For example, age 45 incidence in the South was 26.8/100,000, 36.0% higher than the West, 19.7/100,000 (*p* < 0.0001). Comparing specific registries (Figure 2, Table S1, Spreadsheet S2) reveal more extreme differences. For example, Alaska Native age 45 incidence was 79.9/100,000, 332% higher than San Jose‐Monterey (California), 18.5/100,000 (*p* < 0.0001). Age 42 Kentucky incidence was 19.4/100,000, 111% higher than New Mexico, 9.2/100,000 (*p* < 0.0001). Hawaii had the highest EOCRC IRs at multiple age points (ages 30‐31, 35, 37, 40‐41 and 47) ranging from 75 to 200% higher than the lowest incidence registries.

**Figure 3:**
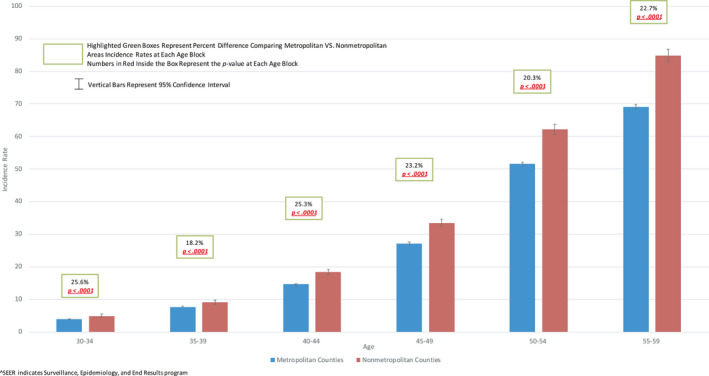
2000 ‐ 2015 Colorectal Cancer Incidence Rates in 5‐Year Age Blocks in Metropolitan VS. Nonmetropolitan Areas in US SEER^ 18, Age 30 – 60 ^SEER indicates Surveillance, Epidemiology, and End Results program

At each 5‐year age block, EOCRC incidence in nonmetropolitan locations was significantly higher than metropolitan (Figure 3). Differences ranged from 18.2 to 25.6% depending on age block. Similar metropolitan/nonmetropolitan disparities were also seen in 50‐ to 54‐ and 55‐ to 59‐year‐olds.

**Figure 4:**
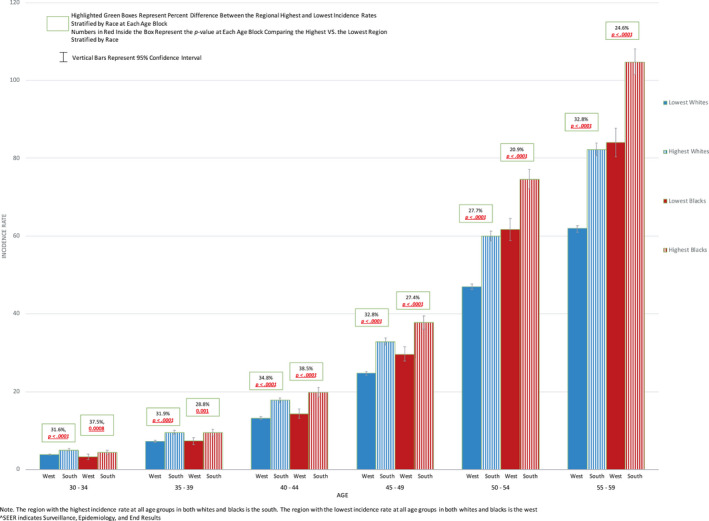
Colorectal Cancer Regional Highest and Lowest Incidence Rates in 5‐Year Age Blocks Stratified by Race in Whites VS. Blacks in US SEER^ 18, Age 30 – 60 Note. The region with the highest incidence rate at all age groups in both Whites and Blacks is the south. The region with the lowest incidence rate at all age groups in both Whites and Blacks is the west. ^SEER indicates Surveillance, Epidemiology, and End Results program

Regional EOCRC stratified by race is depicted in Figure 4. Significant intra‐racial disparities within both White and Black populations are seen. For example, at age 40‐44, the incidence in Blacks in the south is 19.8/100,000, 38.5% higher than the incidence of 14.3/100,000 in the west (*p* < 0.0001). In Whites, for ages 40‐44 the IR in the south is 17.8/100,000, 34.8% higher than the incidence of 13.2/100,000 in the west (*p* < 0.0001).

**Figure 5:**
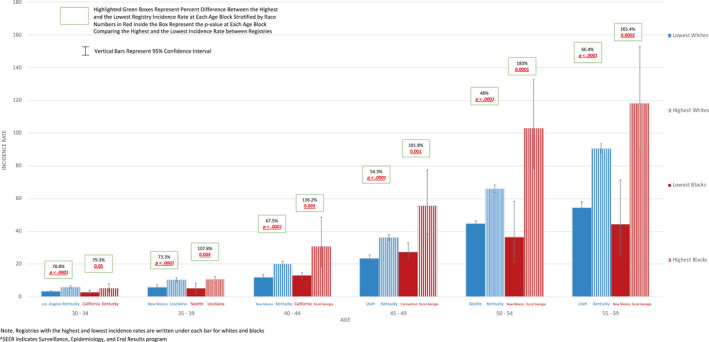
Highest and Lowest Registry Colorectal Cancer Incidence Rates in 5‐Year Age Blocks Stratified by Race in Whites VS. Blacks in US SEER^ 18, Age 30 – 60 Note. Registries with the highest and lowest incidence rates are written under each bar for Whites and Blacks. ^SEER indicates Surveillance, Epidemiology, and End Results program

When individual registries were analyzed, intra‐racial geographic EOCRC disparities are magnified [Figure 5 and Spreadsheet S3 (for 1‐year age increments)]. In Blacks, the most extreme difference between IRs (136.2%) was between rural Georgia (30.7/100,000) and California excluding San Francisco‐Oakland/San Jose‐Monterey/Los Angeles (13/100,000) in 40‐ to 44‐year‐olds (*p* = 0.003). In Whites, the greatest percent difference (78.8%) was between Kentucky (5.9/100,000) and Los Angeles (3.3/100,000) in 30‐ to 34‐year‐olds (*p* < 0.0001).

As above, for combined SEER 18 registries, CRC IRs were higher in nonmetropolitan versus metropolitan locations (Figure 3). When racial stratification was performed, this pattern was also generally seen in both White and Black populations and is depicted in Figures S1 and S2.

## 4 DISCUSSION

Despite rising EOCRC IRs, geographic and geographic intra‐racial disparities in young patients have not been well defined. Our analysis has revealed wide‐regional EOCRC disparities. Differences are magnified between individual registries with Alaska Natives, Hawaii, Louisiana, and Kentucky having disproportionately high EOCRC rates. There are higher EOCRC IRs in nonmetropolitan versus metropolitan communities. Wide intra‐racial geographic EOCRC disparities exist within both White and Black populations. Within the Black population, there were disproportionately high IRs in rural Georgia.

The highest regional EOCRC incidence was seen in the south. Furthermore, Kentucky and Louisiana had high EOCRC burdens with rates more than double those in the lowest incidence registries at multiple age points. Higher EOCRC rates in the south parallel data in older patients in whom higher CRC incidence in many southern states has also been demonstrated.^18^ In older patients, a prior study revealed increased CRC mortality in multiple areas of the southern United States.^19^ Of note, the acute incidence rise from age 49 to 50 in all regions in Figure 1 is felt to be related to screening uptake.^20^

The highest EOCRC incidence by individual registry was seen in Alaska Natives. Incidence was 63.6/100,000 at age 44 and 79.9/100,000 at age 45, more than four times higher than the lowest incidence registries, Utah and San Jose‐Monterey, respectively. Alaska Natives were previously shown to have high EOCRC rates with one study demonstrating an age 40‐49 incidence of 46.1 compared to 14.0/100,000 in Alaskan Whites.^21^ Under age 40 incidence in this study was 2.3/100,000, the same as the White population. Another study demonstrated that 16.5% of American Indians and Alaska Natives with CRC present prior to age 50 compared to 6.7% in Non‐Hispanic Whites.^22^ In 2013, it was recommended Alaska Natives undergo routine screening at age 40.^23^ Our findings reaffirm the marked disparity in this population compared to other EOCRC populations throughout the United States but also demonstrate high under age 40 IRs as well (16.1/100,000 at age 30, 21.9/100,000 at age 36 and 26.0/100,000 at age 37; Spreadsheet S2). It is unlikely these disproportionately high EOCRC rates are due to screening detection given the majority of our study period (2000‐2015) is prior to 2013, the year earlier screening was recommended. Furthermore, high rates were seen prior to age 40.

Disproportionately high EOCRC rates were also found in Hawaii with incidence ranging from 75 to 200% higher than the lowest incidence registries at multiple age points. Racial stratification reveals a trend toward higher rates in Asian/Pacific Islanders, however there is a substantial overlap in confidence intervals (Table S2). A prior analysis revealed that 12.7% of Asian/Pacific Islanders with CRC present prior to age 50 compared to 6.7% in Non‐Hispanic Whites, but this did not focus on Hawaii specifically.^22^ Studies have demonstrated very high rates of CRC in the Japanese population of Hawaii but there are little data on EOCRC.^24^ A recent study demonstrated similar to the rest of the US EOCRC rates in Hawaii have also been increasing.^25^ However, our analysis sheds new light on this population, revealing markedly higher pooled EOCRC rates compared to other US populations.

Our analysis demonstrated wide EOCRC intra‐racial geographic disparities within both White and Black populations. Prior studies have focused on increased early‐onset CRC incidence in Whites and Blacks over time, but to our knowledge ours is the first analysis to focus on pooled incidence rate disparities within racial groups in a recent time period.^26^ A recent study by Siegel et al. focused on where in the US EOCRC rates have been rising, utilizing average annual percent change (APC), in the combined age 20‐49 group.^27^ They demonstrated EOCRC rates continue to rise mainly in the White population, particularly in western states. We demonstrated that despite these increases, there are marked geographic intra‐racial incidence disparities within the Black population, which become most apparent when smaller age blocks and individual registries are compared. For example, in 40‐ to 44‐year‐olds there was a 136.2% intra‐racial incidence rate disparity between rural Georgia and California (excluding San Francisco‐Oakland/San Jose‐Monterey/Los Angeles). Utilization of pooled 2000‐2015 IRs at multiple age points offers important complementary information to APCs because registries with low APCs may have high overall pooled CRC IRs and vice versa.^27^

To our knowledge, this is the first study demonstrating disproportionately high EOCRC rates in the Black population of rural Georgia. A prior study showed higher overall CRC rates in rural versus urban Georgia.^28^ Furthermore, known CRC risk factors such as higher physical inactivity, obesity, and smoking rates were demonstrated in rural versus urban Georgia, but racial and age stratification were not reported.^1^, ^28^

The identification of disparities can assist with two main goals. First, identifying higher risk EOCRC subgroups can allow for community‐based prevention and early detection efforts aimed at large segments of the population. Second, it can guide future research to better understand regions with high and/or increasing EOCRC rates.

Pertaining to the former, with the understanding that risk within populations is heterogeneous and individual patient assessments (for cancer family history, symptoms etc.) are imperative, we need to consider geographic disparities in earlier screening age debates (age 45 versus 50) as higher incidence EOCRC populations could derive disproportionate benefit from earlier screening. In the context of finite CRC‐screening resources, clinical benefits can be maximized in those at higher risk.^29^ Given the ACS recommends average‐risk screening begin at age 45 in all populations and the USMSTF recommends screening Blacks at 45, potential approaches to narrow EOCRC disparities include utilizing these options in general, and especially in higher risk populations.^2^, ^6^ When one considers, for example, the age 45‐49 incidence in Blacks in rural Georgia (55.5/100,000) is 137.2% higher than the age 45‐49 incidence in Whites in Utah (23.4/100,000), we can see how CRC prevention or detection disparities can emerge if the same screening initiation age of 50 was applied to both populations.

Although earlier screening can be recommended, barriers may limit implementation. It is critical to minimize barriers in all populations, especially states and regions with higher EOCRC burdens that often have concurrent higher poverty rates, an important impediment to screening.^30^, ^31^, ^32^, ^33^ Rural communities, which we have demonstrated have higher EOCRC rates, may have lower CRC‐screening rates than urban communities.^9^ Lack of health insurance, increased poverty prevalence, and lower average educational attainment are believed to potentially contribute to rural screening disparities. Even if patients have health insurance, not all carriers cover earlier screening and encouraging them to do so will be beneficial.^34^ EOCRC state registry incidence data can be presented to local insurance carriers so they are aware of the cancer burden in populations they cover. State health agencies can also utilize local incidence data to target higher risk populations and develop modalities to address screening barriers that may be unique to local communities. For example, patient navigation using a biopsychosocial approach is felt to be a modality that can help target health‐care disparities in rural areas.^35^

Narrowing disparities should also include focusing on modifiable risk factors to prevent colonic neoplasia development, particularly given marked EOCRC disparities at very young ages, prior to when average‐risk screening might first be considered. Obesity, for example, may play a role in EOCRC development in general but tends to cluster in states with high EOCRC rates.^1^, ^30^, ^36^ Acting on concerning symptoms earlier can potentially allow cancer detection at an earlier stage. Unfortunately, there are often long delays between symptomatology onset and cancer diagnosis.^37^

Regarding future research, investigative efforts to compare higher EOCRC risk populations to lower risk populations has potential to elucidate factors contributing to cancer development. Assessing differences in exposures and risk factors by race/ethnicity has been proposed as a priority for future research.^38^ A prior study demonstrated no difference in advanced precancerous colorectal neoplasms (largely comprised of advanced polyps, precursor lesions for most CRCs) between Blacks and Whites undergoing average‐risk colonoscopy.^39^ The authors concluded differences in CRC incidence are less likely due to biology and more likely due to behavioral or sociocultural differences in symptom recognition, need for diagnostic evaluation and/or access or uptake of preventive services and the age to initiate screening need not vary based on race alone. Given marked geographic and intra‐racial EOCRC rate disparities we have identified within both White and Black populations at very young ages (i.e., 30‐34, 35‐39, prior to screening age) argues other factors in addition to symptom recognition or screening access may underlie such variations. Future case‐control or prospective studies comparing potential risk factors between populations with the most disparate EOCRC rates could help elucidate such factors.

Study limitations include SEER 18 that only represents 28% of the US population, which could potentially impede the extrapolation of findings, including degree of incidence disparities, to other US areas.^40^ In addition, in some subgroups analyses, lower case counts could potentially limit the interpretation of results, including our analysis in 1‐year age intervals. An advantage of analyzing by 1‐year age intervals is to help assure potentially high‐risk regions and registries at a given age are not masked due to incorporation within a larger patient age analysis block. However, a trade‐off is that statistical power may be limited in some of the smaller subgroups. Furthermore, as the data are ecologic in nature, etiology of high EOCRC IRs cannot be determined. Finally, due to the population‐based nature of the data sets, assessment of EOCRC risk of smaller subpopulations within our defined subgroups was not able to be defined.

Strengths include a large study population (170,434 cases) over 15 years, utilizing high‐quality SEER data that includes unique and underserved populations. In addition, a detailed yearly age incidence assessment of geographic disparities has not been performed, nor has the analysis of geographic intra‐racial EOCRC disparities. Finally, our study provides important data to complement prior APC studies because registries with low APCs may have high overall pooled CRC IRs and vice versa. If APCs alone are utilized to assess EOCRC burden, high‐risk populations may be overlooked.

In conclusion, marked EOCRC IR disparities exist between regions, individual registries and metropolitan/nonmetropolitan populations. Marked geographic intra‐racial disparities also exist within both White and Black populations. Some of the highest EOCRC rates were seen in southern registries, including Kentucky and Louisiana, and in Alaska Natives. Important novel findings include disproportionately increased EOCRC rates in the Hawaiian population and in the Black population of rural Georgia. Future potential investigations include expanding analyses to include all registries of the National Program of Cancer Registries (NPCR) database.^41^ In addition, the high‐risk EOCRC populations we have identified can help guide additional studies to assess etiology.

## CONFLICT OF INTERESTS

Wesal Abualkhair, Meijiao Zhou, Carolina Ochoa, Leonel Lacayo, Caitlin Murphy, and Xiao‐Cheng Wu: None. Jordan J. Karlitz is an Advisor for Exact Sciences, Consultant and Speaker's Bureau for Myriad Genetics, and owens an equity position in Gastro Girl and GI OnDemand.

## AUTHOR CONTRIBUTIONS

Wesal Abualkhair involved in data acquisition, data analysis, data interpretation, literature review, and critical revision of the manuscript. Meijiao Zhou and Xiao‐Cheng Wu involved in data acquisition, data analysis, data interpretation, and critical revision of the manuscript. Carolina Ochoa and Leonel Lacayo involved in literature review and critical revision of the manuscript. Caitlin Murphy involved in Literature review, data interpretation, and critical revision of the manuscript. Jordan J. Karlitz involved in conceptualization and design of the study, drafting of the manuscript, data analysis, data interpretation, and literature review.

## Supporting information

Fig S1

Fig S2

Table S1

Table S2

Supplementary Material

Supplementary Material

Supplementary Material

Supplementary Material

## Data Availability

The dataset analyzed during the current study is available in the Surveillance, Epidemiology, and End Results (SEER) database and can be accessed in detail through utilization of SEER*Stat (https://seer.cancer.gov/data/)
