# Supplementary material for: Geographic and intra‐racial disparities in early‐onset colorectal cancer in the SEER 18 registries of the United States
Source: Cancer Med. 2020 Oct 22;9(23):9150–9. doi: 10.1002/cam4.3488 (PMC7724480; doi:10.1002/cam4.3488)
Supplement: Supplementary file 2 — Fig S2 [file CAM4-9-9150-s002.pdf]

Supplemental Figure 2. 2000 - 2015 Colorectal Cancer Incidence Rates in 5 Year Age Blocks in Metropolitan VS. Nonmetropolitan Areas in Blacks in U.S. SEER^ 18, Age 30 - 60

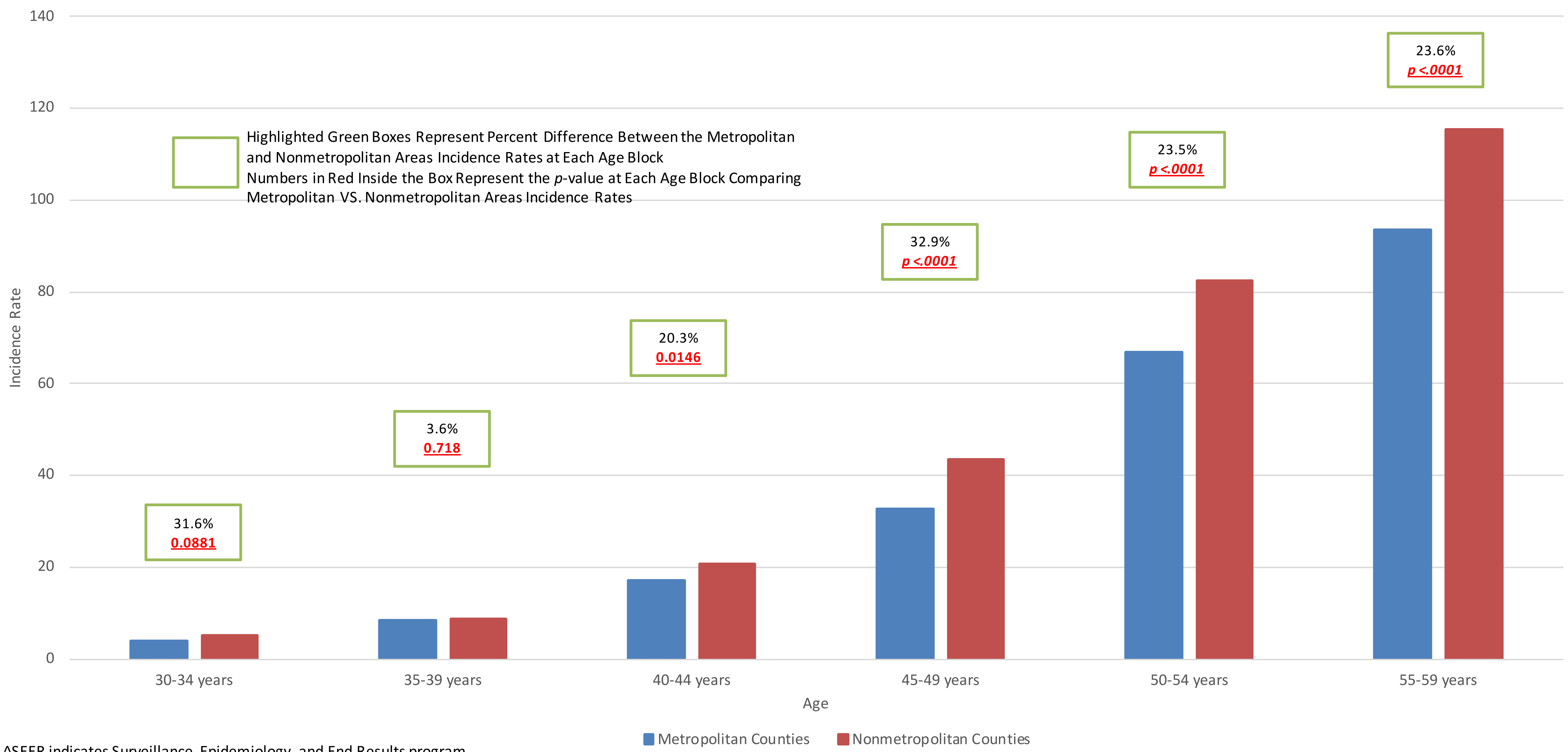

^SEER indicates Surveillance, Epidemiology, and End Results program
