# Supplementary material for: Geographic and intra‐racial disparities in early‐onset colorectal cancer in the SEER 18 registries of the United States
Source: Cancer Med. 2020 Oct 22;9(23):9150–9. doi: 10.1002/cam4.3488 (PMC7724480; doi:10.1002/cam4.3488)
Supplement: Supplementary file 3 — Table S1 [file CAM4-9-9150-s003.docx]

| Supplemental Table 1. Percent Difference Between the Highest and the Lowest Incidence Rates, and the *p*-Value at Each Age Increment | | | | | | |
| --- | --- | --- | --- | --- | --- | --- |
| Age | Lowest Incidence Rates Among SEER^ Registries | Registry Name for Lowest Incidence Rate Among SEER^ Registries | Highest Incidence Rates Among SEER^ Registries | Registry Name for Highest Incidence Rate Among SEER^ Registries | Percent Difference Between the Highest and the Lowest Incidence Rates at Each Age Increment | *p-V*alue at Each Age Increment |
| 30 | 1.7 | Atlanta | 5.1 | Hawaii | 200% | 0.006 |
| 31 | 2.7 | Iowa | 5.3 | Hawaii | 96% | 0.09 |
| 32 | 2.8 | Seattle | 6.3 | Kentucky | 125% | 0.0003 |
| 33 | 3.5 | Iowa | 7.3 | Kentucky | 109% | 0.004 |
| 34 | 3.5 | Utah | 6.9 | Kentucky | 97% | 0.006 |
| 35 | 4.2 | San Francisco-Oakland SMSA | 11.6 | Hawaii | 176% | p <.0001 |
| 36 | 4.7 | Iowa | 9 | Kentucky | 91% | 0.003 |
| 37 | 5.5 | Los Angeles | 10.6 | Hawaii | 93% | 0.003 |
| 38 | 5.3 | San Jose-Monterey | 12.1 | Louisiana | 128% | p <.0001 |
| 39 | 8 | Atlanta | 14.1 | Louisiana | 76% | 0.0001 |
| 40 | 8.7 | New Mexico | 16.1 | Hawaii | 85% | 0.007 |
| 41 | 11.3 | Detroit (Metropolitan) | 19.8 | Hawaii | 75% | 0.001 |
| 42 | 9.2 | New Mexico | 19.4 | Kentucky | 111% | p <.0001 |
| 43 | 13 | New Mexico | 23 | Kentucky | 77% | 0.0001 |
| 44 | 14.2 | Utah | 63.6 | Alaska Natives | 348% | p <.0001 |
| 45 | 18.5 | San Jose-Monterey | 79.9 | Alaska Natives | 332% | p <.0001 |
| 46 | 19.7 | Seattle (Puget Sound) | 31.7 | Kentucky | 61% | p <.0001 |
| 47 | 19.9 | New Mexico | 37.6 | Hawaii | 89% | p <.0001 |
| 48 | 25 | Utah | 69.3 | Alaska Natives | 177% | 0.001 |
| 49 | 25.2 | Utah | 70 | Alaska Natives | 178% | 0.001 |
| 50 | 35.4 | New Mexico | 106 | Alaska Natives | 199% | p <.0001 |
| 51 | 42.5 | Seattle (Puget Sound) | 114.2 | Alaska Natives | 169% | p <.0001 |
| 52 | 44.1 | California excluding San Francisco-Oakland/San Jose-Monterey/Los Angeles | 106.1 | Alaska Natives | 141% | 0.0005 |
| 53 | 45.5 | Seattle (Puget Sound) | 124.4 | Alaska Natives | 173% | p <.0001 |
| 54 | 47.9 | Utah | 114.8 | Alaska Natives | 140% | 0.0007 |
| 55 | 45.7 | Utah | 127.6 | Alaska Natives | 179% | p <.0001 |
| 56 | 49.1 | San Jose-Monterey | 186.3 | Alaska Natives | 279% | p <.0001 |
| 57 | 55.4 | Utah | 99 | Louisiana | 79% | p <.0001 |
| 58 | 51 | Utah | 159.7 | Alaska Natives | 213% | p <.0001 |
| 59 | 64.5 | Utah | 128.1 | Alaska Natives | 99% | 0.01 |
| 60 | 68.5 | Utah | 236.2 | Alaska Natives | 245% | p <.0001 |
| ^SEER indicates Surveillance, Epidemiology, and End Results program | | | | | | |
