# Supplementary material for: Geographic and intra‐racial disparities in early‐onset colorectal cancer in the SEER 18 registries of the United States
Source: Cancer Med. 2020 Oct 22;9(23):9150–9. doi: 10.1002/cam4.3488 (PMC7724480; doi:10.1002/cam4.3488)
Supplement: Supplementary file 4 — Table S2 [file CAM4-9-9150-s004.docx]

| **Supplemental Table 2. Early-Onset Colorectal Cancer Incidence Rates (per 100,000) in Hawaii Stratified by Race** | | | | |
| --- | --- | --- | --- | --- |
|  | Age 30-34 | Age 35-39 | Age 40-44 | Age 45-49 |
| Asian or Pacific Islander | 4.5 (CI 3.3-6.1) | 11.0 (CI 9.0-13.3) | 19.9 (CI 17.3-22.8) | 34.5 (CI 31.1-38.3) |
| White | 3.5 (CI 1.9-6.0) | 9.4 (CI 6.5-13.1) | 17.0 (CI 13.1-21.7) | 30.1 (CI 25.1-35.9) |
| Black | 5.8 (CI 1.2-16.9) | 6.7 (CI 1.4-19.8) | 16.5 (CI 6.0-36.0) | 32.7 (CI 15-62.3) |
| CI=Confidence Interval | | | | |
