# Supplementary material for: Geographic and intra‐racial disparities in early‐onset colorectal cancer in the SEER 18 registries of the United States
Source: Cancer Med. 2020 Oct 22;9(23):9150–9. doi: 10.1002/cam4.3488 (PMC7724480; doi:10.1002/cam4.3488)
Supplement: Supplementary file 5 — Supplementary Material [file CAM4-9-9150-s005.pdf]

Supplemental Spreadsheet 1. Early-Onset Colorectal Cancer Incidence Rates Stratified by U.S. Regions in One Year Age Increments										
Age	30	30	30	30	30	30	30	30	30	30
	Rate	SE	Lower CI	Upper CI	Count	Rate Ratio	Ratio Lower CI	Ratio Upper CI	Ratio P-Value	Pop
US 18	2.8	0.1	2.6	3.1	538					18,911,541
West	2.7	0.2	2.4	3.1	300	0.9579	0.8289	1.1052	0.5758	11,009,415
Northeast	2.8	0.3	2.2	3.6	72	1.0018	0.7722	1.283	1	2,526,451
South	3.1	0.3	2.6	3.7	122	1.0827	0.8821	1.3202	0.4555	3,960,889
Midwest	3.1	0.5	2.3	4.2	44	1.0932	0.785	1.4879	0.6136	1,414,786
Age	31	31	31	31	31	31	31	31	31	31
	Rate	SE	Lower CI	Upper CI	Count	Rate Ratio	Ratio Lower CI	Ratio Upper CI	Ratio P-Value	Pop
US 18	3.3	0.1	3.1	3.6	624					18,714,968
West	3.1	0.2	2.8	3.5	337	0.935	0.8165	1.0691	0.3365	10,810,249
Northeast	3.5	0.4	2.8	4.3	89	1.0569	0.8367	1.3216	0.658	2,525,513
South	3.9	0.3	3.3	4.6	156	1.1828	0.986	1.4118	0.0705	3,955,579
Midwest	3	0.5	2.1	4	42	0.8848	0.6313	1.2101	0.4957	1,423,627
Age	32	32	32	32	32	32	32	32	32	32
	Rate	SE	Lower CI	Upper CI	Count	Rate Ratio	Ratio Lower CI	Ratio Upper CI	Ratio P-Value	Pop
US 18	3.8	0.1	3.5	4.1	719					18,854,636
West	3.5	0.2	3.2	3.9	384	0.9273	0.8171	1.0511	0.2443	10,859,336
Northeast	3.5	0.4	2.8	4.3	91	0.9281	0.7379	1.1556	0.5433	2,571,112
South	4.9#	0.4	4.3	5.7	196	1.29	1.0959	1.5128	0.0023	3,984,191
Midwest	3.3	0.5	2.5	4.4	48	0.8741	0.6385	1.1715	0.4083	1,439,997
Age	33	33	33	33	33	33	33	33	33	33
	Rate	SE	Lower CI	Upper CI	Count	Rate Ratio	Ratio Lower CI	Ratio Upper CI	Ratio P-Value	Pop
US 18	4.9	0.2	4.5	5.2	914					18,811,084
West	4.5	0.2	4.1	4.9	488	0.9295	0.8311	1.0386	0.2013	10,805,177
Northeast	4.9	0.4	4.1	5.8	127	1.0097	0.832	1.2169	0.9467	2,588,576
South	5.9#	0.4	5.1	6.7	233	1.2043	1.0385	1.3921	0.014	3,981,767
Midwest	4.6	0.6	3.6	5.8	66	0.9462	0.7256	1.2155	0.7218	1,435,564
Age	34	34	34	34	34	34	34	34	34	34
	Rate	SE	Lower CI	Upper CI	Count	Rate Ratio	Ratio Lower CI	Ratio Upper CI	Ratio P-Value	Pop
US 18	5.1	0.2	4.8	5.4	954					18,804,219
West	4.6	0.2	4.2	5	490	0.8991	0.8046	1.0036	0.0581	10,742,830
Northeast	5.2	0.4	4.4	6.2	137	1.0259	0.8513	1.2282	0.8071	2,632,348
South	6.0#	0.4	5.2	6.8	238	1.1777	1.0174	1.3588	0.0284	3,983,442
Midwest	6.2	0.7	4.9	7.6	89	1.2135	0.9654	1.5092	0.0969	1,445,599
Age	35	35	35	35	35	35	35	35	35	35
	Rate	SE	Lower CI	Upper CI	Count	Rate Ratio	Ratio Lower CI	Ratio Upper CI	Ratio P-Value	Pop
US 18	5.9	0.2	5.6	6.3	1,120					18,926,176
West	5.5	0.2	5	5.9	591	0.9264	0.8371	1.0244	0.1386	10,779,978
Northeast	6.2	0.5	5.3	7.2	168	1.0504	0.8878	1.2364	0.5755	2,702,623
South	6.8#	0.4	6	7.7	273	1.155	1.0081	1.3195	0.0378	3,994,205
Midwest	6.1	0.6	4.9	7.5	88	1.026	0.8163	1.2754	0.8482	1,449,370

Age	36	36	36	36	36	36	36	36	36	36
	Rate	SE	Lower CI	Upper CI	Count	Rate Ratio	Ratio Lower CI	Ratio Upper CI	Ratio P-Value	Pop
US 18	6.7	0.2	6.4	7.1	1,276					18,944,410
West	6.6	0.2	6.1	7.1	707	0.9786	0.8914	1.0737	0.663	10,725,933
Northeast	6.4	0.5	5.5	7.4	176	0.9534	0.8097	1.1169	0.5841	2,740,790
South	7.9#	0.4	7.1	8.8	318	1.1761	1.0369	1.3309	0.0117	4,014,248
Midwest	5.1#	0.6	4	6.4	75	0.7609	0.5946	0.9608	0.0202	1,463,439
Age	37	37	37	37	37	37	37	37	37	37
	Rate	SE	Lower CI	Upper CI	Count	Rate Ratio	Ratio Lower CI	Ratio Upper CI	Ratio P-Value	Pop
US 18	7.3	0.2	6.9	7.7	1,395					19,035,820
West	6.5#	0.2	6.1	7	703	0.8933	0.8147	0.9788	0.0151	10,738,838
Northeast	8.1	0.5	7.1	9.2	226	1.1073	0.9578	1.2753	0.1685	2,785,076
South	8.8#	0.5	7.9	9.8	356	1.2034	1.0682	1.3529	0.0024	4,036,843
Midwest	7.5	0.7	6.1	9	110	1.0176	0.8303	1.2363	0.8884	1,475,063
Age	38	38	38	38	38	38	38	38	38	38
	Rate	SE	Lower CI	Upper CI	Count	Rate Ratio	Ratio Lower CI	Ratio Upper CI	Ratio P-Value	Pop
US 18	8.9	0.2	8.5	9.3	1,706					19,161,807
West	8.1#	0.3	7.6	8.7	872	0.9099	0.8376	0.9878	0.024	10,764,429
Northeast	9.5	0.6	8.4	10.7	269	1.0661	0.934	1.2131	0.3454	2,834,041
South	10.6#	0.5	9.6	11.7	432	1.1928	1.0708	1.3264	0.0014	4,068,035
Midwest	8.9	0.8	7.4	10.5	133	0.999	0.831	1.1924	1	1,495,302
Age	39	39	39	39	39	39	39	39	39	39
	Rate	SE	Lower CI	Upper CI	Count	Rate Ratio	Ratio Lower CI	Ratio Upper CI	Ratio P-Value	Pop
US 18	10.2	0.2	9.8	10.7	1,977					19,309,878
West	9.4#	0.3	8.9	10	1,019	0.9211	0.8533	0.994	0.0342	10,804,808
Northeast	10.5	0.6	9.4	11.8	304	1.0279	0.9079	1.1603	0.6736	2,888,787
South	12.1#	0.5	11.1	13.2	497	1.184	1.071	1.3071	0.001	4,099,822
Midwest	10.4	0.8	8.8	12.1	157	1.0112	0.854	1.1902	0.9167	1,516,461
Age	40	40	40	40	40	40	40	40	40	40
	Rate	SE	Lower CI	Upper CI	Count	Rate Ratio	Ratio Lower CI	Ratio Upper CI	Ratio P-Value	Pop
US 18	11.8	0.2	11.3	12.3	2,301					19,493,038
West	10.6#	0.3	10	11.2	1,147	0.894	0.8321	0.96	0.0019	10,869,262
Northeast	11.8	0.6	10.6	13.1	347	0.9981	0.889	1.1179	1	2,945,154
South	14.5#	0.6	13.3	15.7	597	1.2244	1.1171	1.3403	0	4,130,644
Midwest	13.6	0.9	11.8	15.5	210	1.1493	0.993	1.3242	0.0619	1,547,978
Age	41	41	41	41	41	41	41	41	41	41
	Rate	SE	Lower CI	Upper CI	Count	Rate Ratio	Ratio Lower CI	Ratio Upper CI	Ratio P-Value	Pop
US 18	13.2	0.3	12.7	13.7	2,583					19,612,049
West	12.1#	0.3	11.5	12.8	1,319	0.9207	0.8609	0.9842	0.0149	10,877,856
Northeast	13	0.7	11.8	14.4	391	0.9907	0.8885	1.1023	0.8902	2,996,534
South	16.2#	0.6	15	17.5	677	1.2332	1.1315	1.3427	0	4,168,169
Midwest	12.5	0.9	10.8	14.4	196	0.9482	0.8158	1.0968	0.4988	1,569,490

Age	42	42	42	42	42	42	42	42	42	42
	Rate	SE	Lower CI	Upper CI	Count	Rate Ratio	Ratio Lower CI	Ratio Upper CI	Ratio P-Value	Pop
US 18	14.5	0.3	14	15	2,858					19,717,232
West	13.3#	0.3	12.6	14	1,449	0.9186	0.8617	0.9789	0.0086	10,882,425
Northeast	14	0.7	12.7	15.4	427	0.969	0.8732	1.0731	0.5635	3,040,183
South	17.3#	0.6	16	18.6	725	1.1916	1.0968	1.2932	0	4,197,555
Midwest	16.1	1	14.2	18.2	257	1.1102	0.9733	1.2617	0.1192	1,597,069
Age	43	43	43	43	43	43	43	43	43	43
	Rate	SE	Lower CI	Upper CI	Count	Rate Ratio	Ratio Lower CI	Ratio Upper CI	Ratio P-Value	Pop
US 18	16.7	0.3	16.2	17.3	3,316					19,828,459
West	15.0#	0.4	14.3	15.8	1,641	0.8987	0.8466	0.9538	0.0004	10,918,361
Northeast	17.9	0.8	16.4	19.4	549	1.0683	0.9743	1.1696	0.1601	3,072,981
South	20.1#	0.7	18.8	21.5	849	1.2043	1.1156	1.299	0	4,215,429
Midwest	17.1	1	15.1	19.2	277	1.0214	0.9003	1.1549	0.753	1,621,688
Age	44	44	44	44	44	44	44	44	44	44
	Rate	SE	Lower CI	Upper CI	Count	Rate Ratio	Ratio Lower CI	Ratio Upper CI	Ratio P-Value	Pop
US 18	18.9	0.3	18.3	19.5	3,733					19,761,829
West	17.2#	0.4	16.4	18	1,864	0.9093	0.8597	0.9615	0.0008	10,852,136
Northeast	19.7	0.8	18.2	21.4	608	1.0444	0.957	1.1382	0.3312	3,081,795
South	22.5#	0.7	21.1	24	943	1.1897	1.1065	1.2781	0	4,196,068
Midwest	19.5	1.1	17.4	21.8	318	1.0316	0.9171	1.1571	0.6103	1,631,830
Age	45	45	45	45	45	45	45	45	45	45
	Rate	SE	Lower CI	Upper CI	Count	Rate Ratio	Ratio Lower CI	Ratio Upper CI	Ratio P-Value	Pop
US 18	21.8	0.3	21.1	22.5	4,284					19,660,161
West	19.7#	0.4	18.8	20.5	2,121	0.903	0.8568	0.9515	0.0001	10,778,977
Northeast	22.7	0.9	21.1	24.5	699	1.044	0.9624	1.1312	0.301	3,072,688
South	26.8#	0.8	25.3	28.5	1,121	1.2321	1.1526	1.3161	0	4,175,502
Midwest	21	1.1	18.8	23.3	343	0.9639	0.861	1.0763	0.5338	1,632,994
Age	46	46	46	46	46	46	46	46	46	46
	Rate	SE	Lower CI	Upper CI	Count	Rate Ratio	Ratio Lower CI	Ratio Upper CI	Ratio P-Value	Pop
US 18	25.1	0.4	24.4	25.8	4,901					19,553,725
West	22.8#	0.5	21.9	23.8	2,442	0.9113	0.8677	0.9568	0.0002	10,691,465
Northeast	25.8	0.9	24	27.6	790	1.0278	0.9522	1.1083	0.4843	3,066,515
South	30.2#	0.9	28.6	32	1,258	1.2062	1.1329	1.2836	0	4,161,026
Midwest	25.1	1.2	22.8	27.7	411	1.0031	0.9048	1.1095	0.9667	1,634,719
Age	47	47	47	47	47	47	47	47	47	47
	Rate	SE	Lower CI	Upper CI	Count	Rate Ratio	Ratio Lower CI	Ratio Upper CI	Ratio P-Value	Pop
US 18	27.5	0.4	26.7	28.2	5,341					19,457,148
West	25.2#	0.5	24.3	26.2	2,678	0.9193	0.8773	0.9632	0.0004	10,612,011
Northeast	27.5	0.9	25.7	29.5	842	1.0031	0.9317	1.0789	0.9451	3,057,976
South	32.5#	0.9	30.8	34.2	1,347	1.1828	1.1134	1.2559	0	4,148,773
Midwest	28.9	1.3	26.4	31.7	474	1.0539	0.9574	1.158	0.2842	1,638,388

Age	48	48	48	48	48	48	48	48	48	48
	Rate	SE	Lower CI	Upper CI	Count	Rate Ratio	Ratio Lower CI	Ratio Upper CI	Ratio P-Value	Pop
US 18	31.3	0.4	30.5	32.1	6,035					19,286,247
West	28.4#	0.5	27.4	29.5	2,983	0.9088	0.8695	0.9497	0	10,489,727
Northeast	32.4	1	30.4	34.4	980	1.034	0.9655	1.1064	0.3402	3,028,850
South	37.3#	1	35.4	39.2	1,538	1.1913	1.1257	1.2601	0	4,125,808
Midwest	32.5	1.4	29.8	35.4	534	1.0394	0.9496	1.1357	0.4039	1,641,862
Age	49	49	49	49	49	49	49	49	49	49
	Rate	SE	Lower CI	Upper CI	Count	Rate Ratio	Ratio Lower CI	Ratio Upper CI	Ratio P-Value	Pop
US 18	34.9	0.4	34.1	35.8	6,689					19,163,643
West	32.0#	0.6	31	33.1	3,337	0.9177	0.8801	0.9567	0	10,418,016
Northeast	34.3	1.1	32.3	36.5	1,033	0.984	0.9207	1.0508	0.6438	3,007,577
South	43.1#	1	41.1	45.1	1,766	1.2343	1.1706	1.3009	0	4,099,140
Midwest	33.7	1.4	31	36.7	553	0.9667	0.8848	1.0544	0.4586	1,638,910
Age	50	50	50	50	50	50	50	50	50	50
	Rate	SE	Lower CI	Upper CI	Count	Rate Ratio	Ratio Lower CI	Ratio Upper CI	Ratio P-Value	Pop
US 18	51	0.5	50	52.1	9,654					18,918,753
West	47.3#	0.7	46	48.7	4,866	0.9271	0.8955	0.9597	0	10,285,607
Northeast	53.5	1.3	50.9	56.2	1,580	1.0476	0.9927	1.105	0.0902	2,955,541
South	58.4#	1.2	56.1	60.8	2,365	1.1447	1.0939	1.1975	0	4,048,850
Midwest	51.8	1.8	48.3	55.4	843	1.0143	0.9442	1.0884	0.7032	1,628,755
Age	51	51	51	51	51	51	51	51	51	51
	Rate	SE	Lower CI	Upper CI	Count	Rate Ratio	Ratio Lower CI	Ratio Upper CI	Ratio P-Value	Pop
US 18	51.5	0.5	50.5	52.6	9,639					18,700,852
West	48.0#	0.7	46.7	49.4	4,874	0.9311	0.8994	0.9639	0	10,155,449
Northeast	52.2	1.3	49.6	54.9	1,524	1.0121	0.9583	1.0684	0.6703	2,921,362
South	60.2#	1.2	57.9	62.7	2,413	1.1688	1.1174	1.2223	0	4,005,249
Midwest	51.1	1.8	47.7	54.8	828	0.9924	0.9233	1.0655	0.8502	1,618,792
Age	52	52	52	52	52	52	52	52	52	52
	Rate	SE	Lower CI	Upper CI	Count	Rate Ratio	Ratio Lower CI	Ratio Upper CI	Ratio P-Value	Pop
US 18	50.6	0.5	49.5	51.6	9,267					18,326,266
West	46.4#	0.7	45.1	47.8	4,614	0.9177	0.8856	0.9507	0	9,943,381
Northeast	51	1.3	48.5	53.7	1,460	1.0095	0.9547	1.0669	0.7451	2,860,076
South	60.8#	1.2	58.4	63.3	2,385	1.2017	1.1483	1.2571	0	3,924,986
Midwest	50.6	1.8	47.1	54.2	808	1	0.9295	1.0747	1	1,597,823
Age	53	53	53	53	53	53	53	53	53	53
	Rate	SE	Lower CI	Upper CI	Count	Rate Ratio	Ratio Lower CI	Ratio Upper CI	Ratio P-Value	Pop
US 18	53.8	0.5	52.7	54.9	9,721					18,073,808
West	48.8#	0.7	47.4	50.2	4,771	0.9066	0.8756	0.9387	0	9,784,106
Northeast	55.7	1.4	53	58.5	1,575	1.0359	0.9816	1.0926	0.1999	2,826,914
South	65.3#	1.3	62.8	67.9	2,536	1.2149	1.1625	1.2693	0	3,880,980
Midwest	53	1.8	49.5	56.8	839	0.9862	0.9179	1.0583	0.715	1,581,808

Age	54	54	54	54	54	54	54	54	54	54
	Rate	SE	Lower CI	Upper CI	Count	Rate Ratio	Ratio Lower CI	Ratio Upper CI	Ratio P-Value	Pop
US 18	58.2	0.6	57.1	59.4	10,245					17,590,040
West	53.5#	0.7	52.1	55	5,096	0.9188	0.8883	0.9503	0	9,522,847
Northeast	56.8	1.4	54	59.7	1,562	0.9751	0.924	1.0286	0.3615	2,750,208
South	72.7#	1.4	70	75.4	2,742	1.2475	1.1956	1.3013	0	3,773,798
Midwest	54.8	1.9	51.1	58.6	845	0.9401	0.8754	1.0086	0.086	1,543,187
Age	55	55	55	55	55	55	55	55	55	55
	Rate	SE	Lower CI	Upper CI	Count	Rate Ratio	Ratio Lower CI	Ratio Upper CI	Ratio P-Value	Pop
US 18	62.1	0.6	60.9	63.3	10,538					16,965,058
West	55.1#	0.8	53.6	56.6	5,054	0.8864	0.857	0.9167	0	9,179,008
Northeast	64.2	1.6	61.1	67.3	1,700	1.0329	0.9807	1.0873	0.2216	2,649,749
South	76.9#	1.5	74.1	79.8	2,804	1.2382	1.1872	1.2909	0	3,645,868
Midwest	65.8	2.1	61.7	70	980	1.0585	0.9905	1.1303	0.0933	1,490,433
Age	56	56	56	56	56	56	56	56	56	56
	Rate	SE	Lower CI	Upper CI	Count	Rate Ratio	Ratio Lower CI	Ratio Upper CI	Ratio P-Value	Pop
US 18	66.4	0.6	65.2	67.7	10,903					16,416,007
West	60.2#	0.8	58.6	61.8	5,340	0.9062	0.8768	0.9364	0	8,872,754
Northeast	68.6	1.6	65.4	71.9	1,757	1.0332	0.9819	1.0867	0.2096	2,560,480
South	80.3#	1.5	77.3	83.3	2,840	1.2084	1.1591	1.2594	0	3,538,637
Midwest	66.9	2.2	62.7	71.2	966	1.0071	0.942	1.0757	0.8417	1,444,136
Age	57	57	57	57	57	57	57	57	57	57
	Rate	SE	Lower CI	Upper CI	Count	Rate Ratio	Ratio Lower CI	Ratio Upper CI	Ratio P-Value	Pop
US 18	70.8	0.7	69.5	72.2	11,238					15,862,397
West	63.7#	0.9	62	65.4	5,453	0.8989	0.8701	0.9285	0	8,562,584
Northeast	72.5	1.7	69.2	75.9	1,798	1.0231	0.9729	1.0754	0.3754	2,480,609
South	87.2#	1.6	84.1	90.4	2,982	1.2305	1.1814	1.2813	0	3,420,645
Midwest	71.9	2.3	67.5	76.4	1,005	1.0143	0.95	1.082	0.6756	1,398,559
Age	58	58	58	58	58	58	58	58	58	58
	Rate	SE	Lower CI	Upper CI	Count	Rate Ratio	Ratio Lower CI	Ratio Upper CI	Ratio P-Value	Pop
US 18	75.6	0.7	74.2	77	11,525					15,244,402
West	67.7#	0.9	65.9	69.5	5,560	0.8954	0.867	0.9246	0	8,213,620
Northeast	78.3	1.8	74.8	81.9	1,865	1.0354	0.9854	1.0874	0.1681	2,382,539
South	92.6#	1.7	89.4	96	3,059	1.2253	1.177	1.2753	0	3,302,189
Midwest	77.3	2.4	72.7	82.2	1,041	1.023	0.9592	1.0901	0.4918	1,346,054
Age	59	59	59	59	59	59	59	59	59	59
	Rate	SE	Lower CI	Upper CI	Count	Rate Ratio	Ratio Lower CI	Ratio Upper CI	Ratio P-Value	Pop
US 18	82.3	0.8	80.8	83.8	11,991					14,571,936
West	75.2#	1	73.2	77.1	5,896	0.9133	0.8851	0.9423	0	7,845,622
Northeast	84.2	1.9	80.5	88.1	1,914	1.0233	0.9746	1.074	0.3549	2,272,934
South	98.0#	1.8	94.6	101.6	3,104	1.1915	1.145	1.2396	0	3,165,892
Midwest	83.7	2.5	78.7	88.8	1,077	1.0166	0.9542	1.0821	0.6144	1,287,488

Age	60	60	60	60	60	60	60	60	60	60
	Rate	SE	Lower CI	Upper CI	Count	Rate Ratio	Ratio Lower CI	Ratio Upper CI	Ratio P-Value	Pop
US 18	89.8	0.8	88.2	91.4	12,449					13,865,681
West	80.9#	1	78.9	83	6,036	0.9015	0.874	0.9297	0	7,457,773
Northeast	92.2	2.1	88.2	96.4	1,995	1.0274	0.9795	1.0772	0.2682	2,162,819
South	109.7#	1.9	106	113.5	3,314	1.2221	1.1758	1.2699	0	3,020,427
Midwest	90.1	2.7	84.9	95.6	1,104	1.0041	0.9433	1.0679	0.9064	1,224,662
	Rates are per 100,000 and age-adjusted to the 2000 US Std Population (single ages to 84 - Census P25-1130) standard									
	Confidence intervals are 95% for rates and ratios.									
#	The rate ratio indicates that the rate is significantly different than the rate for SEER 18 (p<0.05).									
	Warning: Use caution when interpreting ratios and related statistics as the ratio variable contains overlapping groupings.									
