# Supplementary material for: Geographic and intra‐racial disparities in early‐onset colorectal cancer in the SEER 18 registries of the United States
Source: Cancer Med. 2020 Oct 22;9(23):9150–9. doi: 10.1002/cam4.3488 (PMC7724480; doi:10.1002/cam4.3488)
Supplement: Supplementary file 6 — Supplementary Material [file CAM4-9-9150-s006.pdf]

Supplemental Spreadsheet 2. Early-Onset Colorectal Cancer Incidence Rates in SEER 18 Registries in One Year Age Increments										
Age	30	30	30	30	30	30	30	30	30	30
	Rate	SE	Lower CI	Upper CI	Count	Rate Ratio	Ratio Lower CI	Ratio Upper CI	Ratio P-Value	Pop
SEER 18	2.8	0.1	2.6	3.1	538					18,911,541
San Francisco-Oakland SMSA - 2000+	2.8	0.5	1.9	4	31	0.9869	0.6638	1.4176	1	1,104,194
Connecticut - 2000+	2.9	0.6	1.8	4.5	20	1.0166	0.616	1.5859	1	691,537
Detroit (Metropolitan) - 2000+	3.7	0.7	2.5	5.2	30	1.2902	0.862	1.8637	0.2144	817,327
Hawaii - 2000+	5.1#	1.3	2.9	8.4	15	1.7997	1.0003	2.9952	0.0499	292,973
Iowa - 2000+	2.3	0.6	1.3	3.9	14	0.8237	0.4473	1.3942	0.5696	597,459
New Mexico - 2000+	2.2	0.7	1	4.2	9	0.778	0.3538	1.4881	0.5697	406,615
Seattle (Puget Sound) - 2000+	2.4	0.5	1.5	3.5	25	0.8387	0.5379	1.2517	0.4503	1,047,795
Utah - 2000+	3.2	0.7	2	5	21	1.1409	0.7004	1.7619	0.6149	647,033
Atlanta (Metropolitan) - 2000+	1.7	0.4	1	2.9	15	0.609	0.3385	1.0136	0.0576	865,793
San Jose-Monterey - 2000+	2.6	0.6	1.5	4.1	16	0.8972	0.5091	1.4706	0.7853	626,845
Los Angeles - 2000+	2.2	0.3	1.7	2.9	55	0.787	0.5851	1.0399	0.0963	2,456,737
Alaska Natives - 2000+	16.1#	8	4.4	41.2	4	5.655	1.5352	14.5633	0.0121	24,864
Rural Georgia - 2000+	4.4	4.4	0.1	24.4	1	1.5407	0.0389	8.6205	0.9558	22,816
California excluding SF/SJM/LA - 2000+	2.8	0.3	2.3	3.4	124	0.9901	0.8078	1.2057	0.9682	4,402,359
Kentucky - 2000+	4.5#	0.7	3.2	6.1	40	1.5747	1.1123	2.1729	0.0111	892,919
Louisiana - 2000+	3.1	0.6	2.1	4.4	29	1.0874	0.7212	1.5799	0.7138	937,495
New Jersey - 2000+	2.8	0.4	2.1	3.7	52	0.9962	0.7345	1.3258	1	1,834,914
Greater Georgia - 2000+	3	0.5	2.1	4.1	37	1.0473	0.7294	1.4622	0.8339	1,241,866
Age	31	31	31	31	31	31	31	31	31	31
	Rate	SE	Lower CI	Upper CI	Count	Rate Ratio	Ratio Lower CI	Ratio Upper CI	Ratio P-Value	Pop
SEER 18	3.3	0.1	3.1	3.6	624					18,714,968
San Francisco-Oakland SMSA - 2000+	2.7	0.5	1.8	3.8	29	0.8034	0.5336	1.1655	0.2831	1,082,587
Connecticut - 2000+	3	0.7	1.9	4.6	21	0.9117	0.5603	1.406	0.7793	690,816
Detroit (Metropolitan) - 2000+	3.2	0.6	2.1	4.6	26	0.9466	0.6135	1.4004	0.8829	823,756
Hawaii - 2000+	5.3	1.4	3	8.7	15	1.5843	0.8814	2.6335	0.1214	283,959
Iowa - 2000+	2.7	0.7	1.5	4.3	16	0.8	0.4544	1.3095	0.4502	599,871
New Mexico - 2000+	4.2	1	2.5	6.8	17	1.27	0.7351	2.0501	0.3945	401,459
Seattle (Puget Sound) - 2000+	3.4	0.6	2.4	4.7	35	1.0121	0.6985	1.4229	0.9937	1,037,192
Utah - 2000+	3.8	0.8	2.5	5.7	24	1.1512	0.732	1.7289	0.5552	625,262
Atlanta (Metropolitan) - 2000+	3.6	0.6	2.4	5.1	31	1.0771	0.7255	1.5447	0.7378	863,182
San Jose-Monterey - 2000+	2.7	0.7	1.6	4.4	17	0.8242	0.477	1.3304	0.5116	618,610
Los Angeles - 2000+	2.7	0.3	2.1	3.5	65	0.8124	0.6193	1.0501	0.1187	2,399,631
Alaska Natives - 2000+	8.5	6	1	30.6	2	2.5445	0.3075	9.2301	0.3734	23,574
Rural Georgia - 2000+	0	~	0	16	0	0	0	4.8137	0.9278	23,052
California excluding SF/SJM/LA - 2000+	3.1	0.3	2.6	3.6	133	0.9195	0.7567	1.1105	0.407	4,337,975
Kentucky - 2000+	4.1	0.7	2.9	5.7	37	1.2362	0.8622	1.7229	0.2483	897,698
Louisiana - 2000+	4.3	0.7	3.1	5.9	40	1.2924	0.9143	1.7801	0.1451	928,260
New Jersey - 2000+	3.7	0.4	2.9	4.7	68	1.1116	0.8524	1.4295	0.4413	1,834,697
Greater Georgia - 2000+	3.9	0.6	2.8	5.1	48	1.1578	0.8446	1.5541	0.3658	1,243,387

Age	32	32	32	32	32	32	32	32	32	32
	Rate	SE	Lower CI	Upper CI	Count	Rate Ratio	Ratio Lower CI	Ratio Upper CI	Ratio P-Value	Pop
SEER 18	3.8	0.1	3.5	4.1	719					18,854,636
San Francisco-Oakland SMSA - 2000+	3.2	0.5	2.2	4.5	35	0.8394	0.58	1.1785	0.3524	1,093,391
Connecticut - 2000+	3.1	0.7	2	4.7	22	0.8181	0.5095	1.2483	0.4113	705,181
Detroit (Metropolitan) - 2000+	3.1	0.6	2	4.5	26	0.8139	0.528	1.2025	0.347	837,714
Hawaii - 2000+	3.2	1.1	1.4	6	9	0.8266	0.3765	1.5781	0.7088	285,505
Iowa - 2000+	3.7	0.8	2.3	5.5	22	0.9579	0.5965	1.4616	0.9525	602,283
New Mexico - 2000+	3.3	0.9	1.8	5.6	13	0.8632	0.4574	1.4856	0.719	394,937
Seattle (Puget Sound) - 2000+	2.8	0.5	1.9	4	29	0.7268	0.4832	1.053	0.0979	1,046,284
Utah - 2000+	5.5	0.9	3.8	7.6	34	1.4322	0.984	2.02	0.0604	622,546
Atlanta (Metropolitan) - 2000+	3.4	0.6	2.2	4.8	29	0.8806	0.5855	1.2758	0.5726	863,594
San Jose-Monterey - 2000+	3.9	0.8	2.5	5.7	24	1.0133	0.6449	1.5199	1	621,125
Los Angeles - 2000+	2.9#	0.3	2.3	3.7	71	0.7727	0.5968	0.9871	0.0383	2,409,402
Alaska Natives - 2000+	4.3	4.3	0.1	23.7	1	1.1178	0.0283	6.2478	1	23,460
Rural Georgia - 2000+	4.3	4.3	0.1	24.2	1	1.1379	0.0288	6.36	1	23,046
California excluding SF/SJM/LA - 2000+	3.9	0.3	3.3	4.5	168	1.0098	0.8486	1.1961	0.9363	4,362,686
Kentucky - 2000+	6.3#	0.8	4.8	8.2	57	1.654	1.2402	2.1678	0.0008	903,685
Louisiana - 2000+	4.9	0.7	3.6	6.6	46	1.295	0.9395	1.7456	0.1135	931,518
New Jersey - 2000+	3.7	0.4	2.9	4.7	69	0.9697	0.7462	1.2427	0.8687	1,865,931
Greater Georgia - 2000+	5	0.6	3.8	6.4	63	1.3087	0.9951	1.6947	0.0542	1,262,348
Age	33	33	33	33	33	33	33	33	33	33
	Rate	SE	Lower CI	Upper CI	Count	Rate Ratio	Ratio Lower CI	Ratio Upper CI	Ratio P-Value	Pop
SEER 18	4.9	0.2	4.5	5.2	914					18,811,084
San Francisco-Oakland SMSA - 2000+	4.5	0.6	3.3	6	49	0.9296	0.6826	1.2395	0.6822	1,084,827
Connecticut - 2000+	4.8	0.8	3.3	6.7	34	0.9864	0.6789	1.3885	1	709,433
Detroit (Metropolitan) - 2000+	5.3	0.8	3.9	7.1	45	1.0995	0.7963	1.4834	0.5762	842,321
Hawaii - 2000+	5.7	1.4	3.2	9.2	16	1.1638	0.6624	1.9003	0.6165	282,939
Iowa - 2000+	3.5	0.8	2.2	5.4	21	0.7285	0.4488	1.1204	0.1691	593,243
New Mexico - 2000+	4.6	1.1	2.7	7.2	18	0.9368	0.5527	1.489	0.9024	395,451
Seattle (Puget Sound) - 2000+	5.2	0.7	3.9	6.8	54	1.0677	0.7959	1.4055	0.6791	1,040,917
Utah - 2000+	4.2	0.8	2.8	6.2	26	0.8706	0.5656	1.284	0.5581	614,670
Atlanta (Metropolitan) - 2000+	4.9	0.8	3.5	6.6	42	1.0033	0.7181	1.3671	1	861,546
San Jose-Monterey - 2000+	5.5	0.9	3.8	7.7	34	1.1279	0.7763	1.5878	0.538	620,399
Los Angeles - 2000+	4	0.4	3.2	4.9	95	0.8179	0.655	1.0112	0.0642	2,390,638
Alaska Natives - 2000+	8.7	6.1	1.1	31.4	2	1.7868	0.2161	6.4729	0.6169	23,037
Rural Georgia - 2000+	8.7	6.1	1	31.3	2	1.7807	0.2153	6.4508	0.6197	23,116
California excluding SF/SJM/LA - 2000+	4.5	0.3	3.9	5.1	194	0.9174	0.7816	1.0723	0.292	4,352,299
Kentucky - 2000+	7.3#	0.9	5.6	9.2	66	1.4922	1.1442	1.9169	0.0035	910,300
Louisiana - 2000+	5.2	0.8	3.9	6.9	48	1.0772	0.7884	1.4403	0.6568	917,129
New Jersey - 2000+	4.9	0.5	4	6.1	93	1.0186	0.8139	1.2619	0.8974	1,879,143
Greater Georgia - 2000+	5.9	0.7	4.6	7.4	75	1.2157	0.9476	1.5396	0.1238	1,269,676

Age	34	34	34	34	34	34	34	34	34	34
	Rate	SE	Lower CI	Upper CI	Count	Rate Ratio	Ratio Lower CI	Ratio Upper CI	Ratio P-Value	Pop
SEER 18	5.1	0.2	4.8	5.4	954					18,804,219
San Francisco-Oakland SMSA - 2000+	4.1	0.6	3	5.5	44	0.8037	0.5801	1.0874	0.1709	1,079,064
Connecticut - 2000+	5.1	0.8	3.6	7	37	1.008	0.7053	1.3995	1	723,537
Detroit (Metropolitan) - 2000+	6.8#	0.9	5.2	8.8	58	1.3387	1.0087	1.7459	0.0435	853,976
Hawaii - 2000+	2.9	1	1.2	5.7	8	0.5672	0.2442	1.1221	0.1205	278,023
Iowa - 2000+	5.2	0.9	3.6	7.4	31	1.0328	0.6978	1.476	0.9099	591,623
New Mexico - 2000+	6.4	1.3	4.1	9.4	25	1.2617	0.8123	1.8741	0.3001	390,571
Seattle (Puget Sound) - 2000+	4.8	0.7	3.6	6.4	50	0.9525	0.7019	1.2662	0.807	1,034,655
Utah - 2000+	3.5	0.8	2.1	5.3	21	0.682	0.4202	1.0485	0.0866	606,969
Atlanta (Metropolitan) - 2000+	5.2	0.8	3.8	7	45	1.0346	0.7495	1.3953	0.8662	857,336
San Jose-Monterey - 2000+	4.7	0.9	3.1	6.8	29	0.9269	0.6173	1.34	0.7749	616,703
Los Angeles - 2000+	4.1#	0.4	3.3	5	97	0.8047	0.6461	0.9924	0.0418	2,376,122
Alaska Natives - 2000+	8.8	6.2	1.1	31.7	2	1.7293	0.2091	6.2638	0.6441	22,797
Rural Georgia - 2000+	4.2	4.2	0.1	23.4	1	0.8265	0.0209	4.6159	1	23,849
California excluding SF/SJM/LA - 2000+	4.9	0.3	4.3	5.6	214	0.9724	0.8345	1.1289	0.7446	4,337,926
Kentucky - 2000+	6.9#	0.9	5.3	8.8	63	1.3599	1.0366	1.7558	0.0268	913,139
Louisiana - 2000+	6.2	0.8	4.7	8.1	57	1.2265	0.9219	1.603	0.1601	916,043
New Jersey - 2000+	5.2	0.5	4.3	6.4	100	1.0326	0.8318	1.2699	0.7904	1,908,811
Greater Georgia - 2000+	5.7	0.7	4.4	7.1	72	1.1148	0.8648	1.4175	0.4057	1,273,075
Age	35	35	35	35	35	35	35	35	35	35
	Rate	SE	Lower CI	Upper CI	Count	Rate Ratio	Ratio Lower CI	Ratio Upper CI	Ratio P-Value	Pop
SEER 18	5.9	0.2	5.6	6.3	1,120					18,926,176
San Francisco-Oakland SMSA - 2000+	4.2#	0.6	3.1	5.6	46	0.7114	0.5178	0.9554	0.0217	1,092,660
Connecticut - 2000+	6.5	0.9	4.8	8.6	48	1.0907	0.7994	1.4562	0.5962	743,663
Detroit (Metropolitan) - 2000+	5.6	0.8	4.1	7.4	48	0.9453	0.6928	1.2621	0.772	858,034
Hawaii - 2000+	11.6#	2	8	16.3	33	1.9639	1.3451	2.7744	0.0007	283,947
Iowa - 2000+	6.8	1.1	4.8	9.2	40	1.1431	0.8121	1.5665	0.4484	591,336
New Mexico - 2000+	4.3	1	2.5	6.9	17	0.7287	0.423	1.1721	0.2248	394,213
Seattle (Puget Sound) - 2000+	5.3	0.7	4	6.9	55	0.895	0.67	1.1736	0.4662	1,038,400
Utah - 2000+	5.4	1	3.7	7.7	31	0.9205	0.6224	1.3141	0.7311	569,118
Atlanta (Metropolitan) - 2000+	5.2	0.8	3.8	7	45	0.8791	0.6375	1.1842	0.4424	865,034
San Jose-Monterey - 2000+	5.3	0.9	3.7	7.5	33	0.8999	0.6163	1.2713	0.6213	619,690
Los Angeles - 2000+	5.4	0.5	4.5	6.4	129	0.9105	0.7528	1.0933	0.3352	2,394,241
Alaska Natives - 2000+	4.3	4.3	0.1	23.8	1	0.7233	0.0183	4.0384	1	23,362
Rural Georgia - 2000+	0	~	0	15.5	0	0	0	2.6241	0.4897	23,794
California excluding SF/SJM/LA - 2000+	5.6	0.4	5	6.4	246	0.9525	0.8263	1.0944	0.514	4,364,347
Kentucky - 2000+	7.8#	0.9	6.1	9.8	71	1.3119	1.0172	1.6684	0.0367	914,522
Louisiana - 2000+	8.3#	1	6.5	10.4	75	1.4062	1.0978	1.7775	0.0074	901,291
New Jersey - 2000+	6.1	0.6	5.1	7.3	120	1.0351	0.8502	1.2505	0.7469	1,958,960
Greater Georgia - 2000+	6.4	0.7	5.1	7.9	82	1.0745	0.848	1.3454	0.5613	1,289,564

Age	36	36	36	36	36	36	36	36	36	36
	Rate	SE	Lower CI	Upper CI	Count	Rate Ratio	Ratio Lower CI	Ratio Upper CI	Ratio P-Value	Pop
SEER 18	6.7	0.2	6.4	7.1	1,276					18,944,410
San Francisco-Oakland SMSA - 2000+	6.4	0.8	5	8.1	69	0.9475	0.7326	1.2077	0.7186	1,081,157
Connecticut - 2000+	6.3	0.9	4.7	8.4	48	0.9376	0.6877	1.2507	0.728	760,070
Detroit (Metropolitan) - 2000+	5.4	0.8	4	7.2	47	0.8014	0.5858	1.0722	0.1471	870,669
Hawaii - 2000+	8.5	1.7	5.4	12.6	24	1.2597	0.8041	1.883	0.3128	282,854
Iowa - 2000+	4.7	0.9	3.1	6.8	28	0.7013	0.4641	1.0185	0.0641	592,770
New Mexico - 2000+	5.3	1.2	3.3	8.2	21	0.7931	0.4892	1.2175	0.3388	393,140
Seattle (Puget Sound) - 2000+	7.5	0.9	6	9.4	78	1.1188	0.8785	1.4068	0.3652	1,035,061
Utah - 2000+	8.8	1.3	6.5	11.6	49	1.3061	0.9611	1.7374	0.0872	556,986
Atlanta (Metropolitan) - 2000+	6.8	0.9	5.1	8.8	58	1.0054	0.759	1.3082	1	856,526
San Jose-Monterey - 2000+	5	0.9	3.4	7.1	31	0.7455	0.5044	1.0635	0.1136	617,395
Los Angeles - 2000+	5.5#	0.5	4.6	6.6	131	0.8202	0.6799	0.9824	0.0305	2,371,306
Alaska Natives - 2000+	21.9#	9.8	7.1	51.1	5	3.2517	1.054	7.6083	0.0411	22,829
Rural Georgia - 2000+	8.2	5.8	1	29.6	2	1.215	0.147	4.3978	0.9803	24,440
California excluding SF/SJM/LA - 2000+	6.8	0.4	6.1	7.7	299	1.0169	0.8936	1.1543	0.8138	4,365,205
Kentucky - 2000+	9.0#	1	7.2	11.2	83	1.3396	1.0597	1.6735	0.0149	919,851
Louisiana - 2000+	7.7	0.9	6	9.7	70	1.1407	0.8836	1.4515	0.3133	911,080
New Jersey - 2000+	6.5	0.6	5.4	7.7	128	0.9594	0.7937	1.1514	0.6961	1,980,720
Greater Georgia - 2000+	8.1	0.8	6.6	9.8	105	1.197	0.9715	1.4614	0.091	1,302,351
Age	37	37	37	37	37	37	37	37	37	37
	Rate	SE	Lower CI	Upper CI	Count	Rate Ratio	Ratio Lower CI	Ratio Upper CI	Ratio P-Value	Pop
SEER 18	7.3	0.2	6.9	7.7	1,395					19,035,820
San Francisco-Oakland SMSA - 2000+	7.1	0.8	5.6	8.8	76	0.9627	0.7539	1.2132	0.8036	1,077,259
Connecticut - 2000+	6.7	0.9	5	8.8	52	0.9161	0.6808	1.2083	0.5905	774,547
Detroit (Metropolitan) - 2000+	6.3	0.8	4.7	8.1	55	0.8535	0.6396	1.1175	0.2738	879,375
Hawaii - 2000+	10.6	1.9	7.2	15.1	30	1.4469	0.9725	2.075	0.0679	282,928
Iowa - 2000+	9.2	1.2	7	12	55	1.2599	0.9442	1.6497	0.1154	595,688
New Mexico - 2000+	8.6	1.5	5.9	12	34	1.1674	0.8052	1.6392	0.4177	397,426
Seattle (Puget Sound) - 2000+	5.9	0.8	4.6	7.6	62	0.8113	0.6186	1.0465	0.1135	1,042,796
Utah - 2000+	6.1	1.1	4.2	8.5	33	0.8263	0.5665	1.166	0.3146	544,959
Atlanta (Metropolitan) - 2000+	8.4	1	6.6	10.6	72	1.1488	0.8936	1.4564	0.2793	855,221
San Jose-Monterey - 2000+	7.3	1.1	5.3	9.7	45	0.9938	0.7215	1.3369	1	617,915
Los Angeles - 2000+	5.5#	0.5	4.6	6.5	129	0.7451	0.6172	0.8929	0.0011	2,362,509
Alaska Natives - 2000+	26.0#	10.6	9.6	56.7	6	3.553	1.3016	7.7529	0.0157	23,044
Rural Georgia - 2000+	8.2	5.8	1	29.5	2	1.1128	0.1346	4.0275	1	24,524
California excluding SF/SJM/LA - 2000+	6.6	0.4	5.8	7.4	288	0.8952	0.7858	1.017	0.0906	4,390,002
Kentucky - 2000+	9.1	1	7.2	11.2	84	1.235	0.9788	1.5399	0.0748	928,143
Louisiana - 2000+	9.4#	1	7.5	11.6	86	1.2839	1.0204	1.597	0.0332	914,018
New Jersey - 2000+	8.7#	0.7	7.4	10	174	1.181	1.0029	1.3834	0.0461	2,010,529
Greater Georgia - 2000+	8.5	0.8	7	10.2	112	1.1623	0.9501	1.4098	0.1433	1,314,937

Age	38	38	38	38	38	38	38	38	38	38
	Rate	SE	Lower CI	Upper CI	Count	Rate Ratio	Ratio Lower CI	Ratio Upper CI	Ratio P-Value	Pop
SEER 18	8.9	0.2	8.5	9.3	1,706					19,161,807
San Francisco-Oakland SMSA - 2000+	8.8	0.9	7.1	10.8	95	0.9904	0.7969	1.2181	0.9812	1,077,407
Connecticut - 2000+	8.9	1.1	6.9	11.2	70	0.9944	0.7715	1.2631	1	790,706
Detroit (Metropolitan) - 2000+	9.3	1	7.4	11.6	83	1.0468	0.8295	1.3052	0.7152	890,567
Hawaii - 2000+	8.8	1.8	5.7	13	25	0.9914	0.6397	1.4686	1	283,236
Iowa - 2000+	8.3	1.2	6.1	10.9	50	0.9287	0.6865	1.23	0.6679	604,735
New Mexico - 2000+	7.2	1.3	4.8	10.4	29	0.8102	0.5409	1.1679	0.2959	402,045
Seattle (Puget Sound) - 2000+	8.2	0.9	6.6	10.2	86	0.9259	0.7367	1.1501	0.5258	1,043,304
Utah - 2000+	8.4	1.3	6.1	11.3	45	0.9458	0.6873	1.2712	0.7842	534,380
Atlanta (Metropolitan) - 2000+	8.6	1	6.8	10.8	74	0.97	0.7579	1.2244	0.8563	856,909
San Jose-Monterey - 2000+	5.3#	0.9	3.7	7.5	33	0.5992	0.4111	0.8449	0.0022	618,536
Los Angeles - 2000+	7.8	0.6	6.7	9	184	0.8759	0.7482	1.0204	0.091	2,359,443
Alaska Natives - 2000+	17.5	8.8	4.8	44.9	4	1.9682	0.5357	5.0486	0.2982	22,827
Rural Georgia - 2000+	7.9	5.6	1	28.4	2	0.8825	0.1068	3.1928	1	25,455
California excluding SF/SJM/LA - 2000+	8.4	0.4	7.6	9.3	371	0.9421	0.8398	1.0546	0.3107	4,423,251
Kentucky - 2000+	11.5#	1.1	9.4	13.9	108	1.2926	1.0541	1.5707	0.014	938,490
Louisiana - 2000+	12.1#	1.1	9.9	14.5	111	1.3561	1.1089	1.6438	0.0033	919,392
New Jersey - 2000+	9.7	0.7	8.4	11.2	199	1.0939	0.9397	1.2676	0.2476	2,043,335
Greater Georgia - 2000+	10.3	0.9	8.7	12.2	137	1.1589	0.9666	1.3799	0.1108	1,327,789
Age	39	39	39	39	39	39	39	39	39	39
	Rate	SE	Lower CI	Upper CI	Count	Rate Ratio	Ratio Lower CI	Ratio Upper CI	Ratio P-Value	Pop
SEER 18	10.2	0.2	9.8	10.7	1,977					19,309,878
San Francisco-Oakland SMSA - 2000+	9.3	0.9	7.5	11.3	100	0.9042	0.7321	1.1057	0.3512	1,080,204
Connecticut - 2000+	12.5	1.2	10.2	15.2	102	1.2256	0.9944	1.496	0.0564	812,856
Detroit (Metropolitan) - 2000+	9.7	1	7.8	12	88	0.9515	0.7596	1.1782	0.6966	903,371
Hawaii - 2000+	13.3	2.2	9.4	18.2	38	1.2951	0.9137	1.784	0.1445	286,582
Iowa - 2000+	11.3	1.4	8.8	14.2	69	1.0993	0.8518	1.3976	0.4721	613,090
New Mexico - 2000+	8.5	1.4	5.9	11.9	35	0.8327	0.5783	1.1621	0.3183	410,544
Seattle (Puget Sound) - 2000+	9	0.9	7.3	11	95	0.8801	0.7087	1.0816	0.2408	1,054,280
Utah - 2000+	10.9	1.4	8.3	14.1	57	1.0652	0.8037	1.3859	0.6752	522,674
Atlanta (Metropolitan) - 2000+	8.0#	1	6.2	10.1	68	0.777	0.6009	0.9895	0.0401	854,840
San Jose-Monterey - 2000+	11.5	1.4	9	14.5	71	1.1256	0.8755	1.4265	0.3575	616,074
Los Angeles - 2000+	9.9	0.7	8.7	11.3	234	0.9713	0.8446	1.1128	0.7048	2,352,985
Alaska Natives - 2000+	17.3	8.7	4.7	44.4	4	1.6931	0.4608	4.3418	0.4276	23,076
Rural Georgia - 2000+	11.7	6.8	2.4	34.3	3	1.1474	0.2364	3.3582	0.9709	25,537
California excluding SF/SJM/LA - 2000+	8.6#	0.4	7.8	9.5	385	0.8434	0.7542	0.9412	0.0021	4,458,389
Kentucky - 2000+	13.2#	1.2	11	15.7	125	1.2868	1.0652	1.5423	0.0093	948,828
Louisiana - 2000+	14.1#	1.2	11.7	16.7	130	1.3733	1.141	1.6406	0.0009	924,588
New Jersey - 2000+	9.7	0.7	8.4	11.2	202	0.9504	0.8182	1.099	0.5177	2,075,931
Greater Georgia - 2000+	12.7#	1	10.9	14.8	171	1.2408	1.0551	1.4513	0.0094	1,346,029

Age	40	40	40	40	40	40	40	40	40	40
	Rate	SE	Lower CI	Upper CI	Count	Rate Ratio	Ratio Lower CI	Ratio Upper CI	Ratio P-Value	Pop
SEER 18	11.8	0.2	11.3	12.3	2,301					19,493,038
San Francisco-Oakland SMSA - 2000+	10.6	1	8.8	12.7	116	0.8994	0.7399	1.0842	0.284	1,092,578
Connecticut - 2000+	12.9	1.2	10.5	15.5	107	1.089	0.8885	1.3222	0.4144	832,406
Detroit (Metropolitan) - 2000+	12.7	1.2	10.5	15.3	117	1.0783	0.8877	1.2988	0.4514	919,208
Hawaii - 2000+	16.1	2.3	11.8	21.4	47	1.3618	0.9977	1.817	0.0517	292,386
Iowa - 2000+	14.8#	1.5	11.9	18.1	93	1.253	1.0072	1.542	0.043	628,770
New Mexico - 2000+	8.7	1.4	6.1	12	36	0.7368	0.5147	1.0231	0.0705	413,927
Seattle (Puget Sound) - 2000+	9.8	1	8	11.9	104	0.829	0.6744	1.0092	0.0625	1,062,821
Utah - 2000+	10.9	1.5	8.2	14.1	56	0.9215	0.6939	1.2009	0.5995	514,837
Atlanta (Metropolitan) - 2000+	14.4#	1.3	11.9	17.1	123	1.2173	1.0069	1.4598	0.0423	856,026
San Jose-Monterey - 2000+	9.1	1.2	6.9	11.8	56	0.7693	0.5793	1.0026	0.0525	616,661
Los Angeles - 2000+	9.8#	0.6	8.6	11.1	231	0.8287	0.7207	0.9492	0.006	2,361,340
Alaska Natives - 2000+	43.4#	13.7	20.8	79.8	10	3.6751	1.76	6.771	0.0011	23,051
Rural Georgia - 2000+	19	8.5	6.2	44.4	5	1.6136	0.5234	3.771	0.4039	26,251
California excluding SF/SJM/LA - 2000+	10.9	0.5	10	11.9	491	0.9261	0.8384	1.0213	0.1264	4,491,661
Kentucky - 2000+	15.7#	1.3	13.2	18.4	150	1.3261	1.1166	1.5648	0.0014	958,225
Louisiana - 2000+	14.8#	1.3	12.4	17.4	138	1.2509	1.0457	1.4858	0.0147	934,601
New Jersey - 2000+	11.4	0.7	10	12.9	240	0.9623	0.839	1.0996	0.5988	2,112,748
Greater Georgia - 2000+	13.4	1	11.5	15.4	181	1.1312	0.9669	1.3165	0.1233	1,355,541
Age	41	41	41	41	41	41	41	41	41	41
	Rate	SE	Lower CI	Upper CI	Count	Rate Ratio	Ratio Lower CI	Ratio Upper CI	Ratio P-Value	Pop
SEER 18	13.2	0.3	12.7	13.7	2,583					19,612,049
San Francisco-Oakland SMSA - 2000+	11.3	1	9.4	13.5	122	0.858	0.7096	1.0291	0.1023	1,079,615
Connecticut - 2000+	11.6	1.2	9.4	14.1	99	0.8818	0.714	1.078	0.2351	852,437
Detroit (Metropolitan) - 2000+	11.3	1.1	9.2	13.6	105	0.8553	0.6968	1.0398	0.1221	932,145
Hawaii - 2000+	19.8#	2.6	15.1	25.7	58	1.5067	1.1408	1.9542	0.0044	292,272
Iowa - 2000+	14.3	1.5	11.5	17.5	91	1.0841	0.8697	1.3363	0.4773	637,345
New Mexico - 2000+	11.9	1.7	8.8	15.7	50	0.9023	0.6679	1.1932	0.5221	420,754
Seattle (Puget Sound) - 2000+	11.3	1	9.4	13.5	121	0.8567	0.7079	1.0283	0.1002	1,072,400
Utah - 2000+	12.4	1.6	9.5	15.8	63	0.9406	0.7206	1.2075	0.6875	508,562
Atlanta (Metropolitan) - 2000+	13.7	1.3	11.3	16.4	117	1.0407	0.8572	1.2528	0.699	853,597
San Jose-Monterey - 2000+	11.7	1.4	9.2	14.8	72	0.892	0.6957	1.1274	0.3696	612,855
Los Angeles - 2000+	12.5	0.7	11.1	14	293	0.9468	0.8362	1.0688	0.3925	2,349,733
Alaska Natives - 2000+	21.8	9.8	7.1	51	5	1.6587	0.5381	3.8758	0.3749	22,888
Rural Georgia - 2000+	14.8	7.4	4	38	4	1.1257	0.3065	2.8857	0.9501	26,980
California excluding SF/SJM/LA - 2000+	11.8#	0.5	10.9	12.9	535	0.8989	0.8175	0.987	0.025	4,518,777
Kentucky - 2000+	17.0#	1.3	14.5	19.9	165	1.2946	1.0993	1.5157	0.0022	967,750
Louisiana - 2000+	15.8#	1.3	13.3	18.5	150	1.1975	1.0089	1.4122	0.0394	951,092
New Jersey - 2000+	13.6	0.8	12.1	15.3	292	1.034	0.913	1.1675	0.6051	2,144,097
Greater Georgia - 2000+	17.6#	1.1	15.5	20	241	1.3369	1.1667	1.5261	0	1,368,750

Age	42	42	42	42	42	42	42	42	42	42
	Rate	SE	Lower CI	Upper CI	Count	Rate Ratio	Ratio Lower CI	Ratio Upper CI	Ratio P-Value	Pop
SEER 18	14.5	0.3	14	15	2,858					19,717,232
San Francisco-Oakland SMSA - 2000+	12.8	1.1	10.7	15.1	138	0.8814	0.7375	1.0458	0.155	1,080,222
Connecticut - 2000+	14.3	1.3	11.9	17	124	0.9849	0.8161	1.1791	0.9148	868,619
Detroit (Metropolitan) - 2000+	17	1.3	14.5	19.8	161	1.1717	0.9934	1.3737	0.0598	947,958
Hawaii - 2000+	19.3#	2.6	14.6	25.1	56	1.3345	1.0055	1.738	0.0458	289,506
Iowa - 2000+	14.8	1.5	12	18.1	96	1.0203	0.8237	1.2506	0.8749	649,111
New Mexico - 2000+	9.2#	1.5	6.5	12.6	39	0.6349	0.4505	0.8701	0.0032	423,771
Seattle (Puget Sound) - 2000+	13.8	1.1	11.7	16.2	149	0.9506	0.8008	1.1211	0.5801	1,081,343
Utah - 2000+	12.9	1.6	10	16.5	65	0.8926	0.687	1.1412	0.3998	502,408
Atlanta (Metropolitan) - 2000+	15.9	1.4	13.4	18.9	135	1.1	0.9186	1.3076	0.3003	846,695
San Jose-Monterey - 2000+	15.6	1.6	12.6	19	95	1.0743	0.8663	1.3182	0.5196	610,051
Los Angeles - 2000+	13	0.7	11.6	14.6	305	0.9001	0.7972	1.0132	0.0827	2,337,785
Alaska Natives - 2000+	25.5	10.4	9.4	55.6	6	1.761	0.6457	3.8377	0.2613	23,506
Rural Georgia - 2000+	14.7	7.4	4	37.7	4	1.0153	0.2764	2.6024	1	27,180
California excluding SF/SJM/LA - 2000+	13.1#	0.5	12.1	14.2	596	0.9069	0.8289	0.9909	0.0303	4,533,833
Kentucky - 2000+	19.4#	1.4	16.8	22.4	191	1.3407	1.1519	1.5527	0.0002	982,853
Louisiana - 2000+	15.9	1.3	13.5	18.6	153	1.095	0.9245	1.2888	0.2937	963,936
New Jersey - 2000+	14	0.8	12.4	15.6	303	0.9626	0.8523	1.084	0.5513	2,171,564
Greater Georgia - 2000+	17.6#	1.1	15.4	19.9	242	1.2125	1.059	1.383	0.0055	1,376,891
Age	43	43	43	43	43	43	43	43	43	43
	Rate	SE	Lower CI	Upper CI	Count	Rate Ratio	Ratio Lower CI	Ratio Upper CI	Ratio P-Value	Pop
SEER 18	16.7	0.3	16.2	17.3	3,316					19,828,459
San Francisco-Oakland SMSA - 2000+	15.6	1.2	13.3	18.1	168	0.9319	0.7933	1.0884	0.3949	1,077,990
Connecticut - 2000+	18.3	1.4	15.6	21.3	162	1.0943	0.9288	1.2815	0.2816	885,212
Detroit (Metropolitan) - 2000+	15.5	1.3	13.1	18.2	149	0.9263	0.7807	1.0917	0.3833	961,899
Hawaii - 2000+	19.4	2.6	14.7	25.1	57	1.1582	0.8753	1.5044	0.3038	294,283
Iowa - 2000+	19.4	1.7	16.2	23.1	128	1.1601	0.9646	1.3843	0.1142	659,789
New Mexico - 2000+	13	1.7	9.8	16.9	56	0.7761	0.585	1.0104	0.0606	431,451
Seattle (Puget Sound) - 2000+	13.9#	1.1	11.8	16.3	152	0.8324	0.7028	0.9795	0.0262	1,091,950
Utah - 2000+	14.8	1.7	11.6	18.6	74	0.8851	0.6932	1.1143	0.3253	499,940
Atlanta (Metropolitan) - 2000+	18	1.5	15.2	21.1	151	1.074	0.9063	1.2646	0.4123	840,698
San Jose-Monterey - 2000+	16.2	1.6	13.2	19.7	99	0.9694	0.7856	1.184	0.8103	610,692
Los Angeles - 2000+	14.4#	0.8	12.9	16	334	0.8585	0.7649	0.961	0.0074	2,326,329
Alaska Natives - 2000+	29.8	11.3	12	61.3	7	1.78	0.7151	3.6715	0.208	23,516
Rural Georgia - 2000+	10.9	6.3	2.2	31.7	3	0.6495	0.1339	1.8997	0.6455	27,621
California excluding SF/SJM/LA - 2000+	15.2#	0.6	14.1	16.4	694	0.9096	0.837	0.9875	0.0233	4,562,210
Kentucky - 2000+	23.0#	1.5	20.1	26.2	228	1.3775	1.1992	1.5758	0	989,719
Louisiana - 2000+	20.2#	1.4	17.4	23.2	197	1.2055	1.0387	1.3921	0.0142	977,217
New Jersey - 2000+	17.7	0.9	16	19.5	387	1.0578	0.9496	1.1755	0.3088	2,187,769
Greater Georgia - 2000+	19.6#	1.2	17.3	22	270	1.1698	1.0295	1.3246	0.0164	1,380,174

Age	44	44	44	44	44	44	44	44	44	44
	Rate	SE	Lower CI	Upper CI	Count	Rate Ratio	Ratio Lower CI	Ratio Upper CI	Ratio P-Value	Pop
SEER 18	18.9	0.3	18.3	19.5	3,733					19,761,829
San Francisco-Oakland SMSA - 2000+	19.6	1.4	17.1	22.5	210	1.0393	0.9001	1.1946	0.6063	1,069,670
Connecticut - 2000+	18.9	1.5	16.2	22	169	1.0017	0.8535	1.1689	1	893,157
Detroit (Metropolitan) - 2000+	17.5	1.3	15	20.4	169	0.9281	0.7908	1.083	0.3633	963,967
Hawaii - 2000+	22.6	2.8	17.5	28.6	67	1.1938	0.9232	1.5198	0.1754	297,104
Iowa - 2000+	22.3	1.8	18.9	26.2	149	1.181	0.9959	1.3914	0.0558	667,863
New Mexico - 2000+	17.7	2	13.9	22.1	76	0.9361	0.7359	1.1747	0.6166	429,798
Seattle (Puget Sound) - 2000+	17.2	1.3	14.8	19.8	187	0.9095	0.781	1.0536	0.2161	1,088,492
Utah - 2000+	14.2#	1.7	11.1	18	70	0.7533	0.5859	0.954	0.0171	491,952
Atlanta (Metropolitan) - 2000+	19.3	1.5	16.4	22.5	159	1.0193	0.8641	1.1948	0.8363	825,820
San Jose-Monterey - 2000+	18.7	1.8	15.4	22.5	113	0.9899	0.8135	1.1937	0.9646	604,333
Los Angeles - 2000+	16.0#	0.8	14.4	17.8	368	0.8484	0.7602	0.9446	0.0023	2,296,119
Alaska Natives - 2000+	63.6#	16.4	35.6	104.9	15	3.3663	1.8822	5.5594	0.0001	23,589
Rural Georgia - 2000+	35.3	11.2	16.9	64.9	10	1.8668	0.8945	3.4371	0.0937	28,357
California excluding SF/SJM/LA - 2000+	16.7#	0.6	15.5	17.9	758	0.8817	0.8144	0.9536	0.0015	4,551,079
Kentucky - 2000+	25.2#	1.6	22.1	28.5	249	1.3326	1.1675	1.5154	0	989,147
Louisiana - 2000+	21.8	1.5	18.9	24.9	213	1.152	0.9987	1.3229	0.0521	978,773
New Jersey - 2000+	20.1	1	18.2	22	439	1.0618	0.9597	1.1725	0.2455	2,188,638
Greater Georgia - 2000+	22.7#	1.3	20.3	25.4	312	1.2021	1.0675	1.3497	0.0025	1,373,971
Age	45	45	45	45	45	45	45	45	45	45
	Rate	SE	Lower CI	Upper CI	Count	Rate Ratio	Ratio Lower CI	Ratio Upper CI	Ratio P-Value	Pop
SEER 18	21.8	0.3	21.1	22.5	4,284					19,660,161
San Francisco-Oakland SMSA - 2000+	19	1.3	16.4	21.8	202	0.8703	0.752	1.0024	0.0541	1,065,219
Connecticut - 2000+	21.8	1.6	18.9	25.1	195	1.0022	0.8637	1.1571	0.9962	892,914
Detroit (Metropolitan) - 2000+	21.1	1.5	18.3	24.2	203	0.9693	0.8379	1.1161	0.6971	961,083
Hawaii - 2000+	24.4	2.9	19.2	30.7	73	1.122	0.8778	1.4138	0.3591	298,585
Iowa - 2000+	20.8	1.8	17.5	24.6	140	0.9562	0.8022	1.1317	0.6389	671,911
New Mexico - 2000+	23	2.3	18.7	28	100	1.0568	0.8579	1.2885	0.6125	434,267
Seattle (Puget Sound) - 2000+	20.7	1.4	18	23.5	224	0.9479	0.825	1.0844	0.4577	1,084,467
Utah - 2000+	19.2	2	15.5	23.5	93	0.8791	0.708	1.0796	0.2349	485,488
Atlanta (Metropolitan) - 2000+	22.7	1.7	19.5	26.2	183	1.0404	0.8924	1.2065	0.6203	807,202
San Jose-Monterey - 2000+	18.5	1.8	15.2	22.4	110	0.8512	0.6979	1.0287	0.0989	593,041
Los Angeles - 2000+	19.6#	0.9	17.8	21.5	445	0.8993	0.8138	0.9917	0.033	2,270,942
Alaska Natives - 2000+	79.9#	18.3	48.1	124.8	19	3.6675	2.2059	5.7344	0	23,775
Rural Georgia - 2000+	35.4	11.2	17	65.1	10	1.6237	0.7781	2.989	0.1911	28,264
California excluding SF/SJM/LA - 2000+	18.9#	0.6	17.7	20.2	855	0.8675	0.8051	0.9338	0.0001	4,523,193
Kentucky - 2000+	30.3#	1.7	26.9	33.9	301	1.3892	1.2318	1.5618	0	994,368
Louisiana - 2000+	25.5#	1.6	22.4	28.8	251	1.1686	1.0248	1.3275	0.0202	985,693
New Jersey - 2000+	23.1	1	21.1	25.2	504	1.0611	0.9656	1.164	0.2179	2,179,774
Greater Georgia - 2000+	27.6#	1.4	24.9	30.6	376	1.2688	1.1388	1.4102	0	1,359,975

Age	46	46	46	46	46	46	46	46	46	46
	Rate	SE	Lower CI	Upper CI	Count	Rate Ratio	Ratio Lower CI	Ratio Upper CI	Ratio P-Value	Pop
SEER 18	25.1	0.4	24.4	25.8	4,901					19,553,725
San Francisco-Oakland SMSA - 2000+	23.3	1.5	20.4	26.4	245	0.9285	0.8134	1.0558	0.2697	1,052,735
Connecticut - 2000+	25.1	1.7	22	28.7	225	1.0034	0.874	1.147	0.9794	894,663
Detroit (Metropolitan) - 2000+	24.4	1.6	21.4	27.7	234	0.973	0.8497	1.1096	0.7134	959,501
Hawaii - 2000+	28.8	3.1	23.1	35.6	86	1.1498	0.918	1.4229	0.2234	298,425
Iowa - 2000+	26.2	2	22.5	30.4	177	1.0459	0.8951	1.2152	0.5785	675,218
New Mexico - 2000+	24.6	2.4	20.2	29.7	107	0.9819	0.803	1.1892	0.902	434,766
Seattle (Puget Sound) - 2000+	19.7#	1.3	17.1	22.5	213	0.7849	0.6811	0.9005	0.0004	1,082,699
Utah - 2000+	22.5	2.2	18.4	27.2	108	0.8973	0.7345	1.0858	0.2842	480,211
Atlanta (Metropolitan) - 2000+	26.7	1.8	23.2	30.5	212	1.0637	0.9226	1.2206	0.3969	795,203
San Jose-Monterey - 2000+	21.5	1.9	17.9	25.6	127	0.8586	0.7142	1.0241	0.0929	590,129
Los Angeles - 2000+	23.4	1	21.4	25.5	523	0.9328	0.8508	1.0211	0.1344	2,236,897
Alaska Natives - 2000+	59.3#	15.8	32.4	99.5	14	2.3651	1.2921	3.9721	0.0065	23,617
Rural Georgia - 2000+	49.4#	13.2	27	82.8	14	1.9701	1.0763	3.3087	0.029	28,352
California excluding SF/SJM/LA - 2000+	22.7#	0.7	21.3	24.1	1,019	0.9051	0.8452	0.9685	0.0036	4,491,986
Kentucky - 2000+	31.7#	1.8	28.3	35.4	315	1.263	1.1235	1.4157	0.0001	995,039
Louisiana - 2000+	30.6#	1.8	27.3	34.3	303	1.2211	1.0838	1.3716	0.0011	989,986
New Jersey - 2000+	26	1.1	23.9	28.3	565	1.0379	0.9497	1.1326	0.4138	2,171,852
Greater Georgia - 2000+	30.6#	1.5	27.7	33.7	414	1.2213	1.1021	1.3505	0.0002	1,352,446
Age	47	47	47	47	47	47	47	47	47	47
	Rate	SE	Lower CI	Upper CI	Count	Rate Ratio	Ratio Lower CI	Ratio Upper CI	Ratio P-Value	Pop
SEER 18	27.5	0.4	26.7	28.2	5,341					19,457,148
San Francisco-Oakland SMSA - 2000+	24.3	1.5	21.4	27.5	254	0.8843	0.7766	1.0031	0.0561	1,046,382
Connecticut - 2000+	26.3	1.7	23.1	29.9	236	0.9599	0.839	1.0938	0.5647	895,643
Detroit (Metropolitan) - 2000+	28.8	1.7	25.5	32.4	276	1.0487	0.9258	1.1838	0.4574	958,771
Hawaii - 2000+	37.6#	3.6	31	45.3	112	1.3704	1.1262	1.6524	0.0019	297,741
Iowa - 2000+	29.1	2.1	25.2	33.5	198	1.0613	0.9163	1.2233	0.4295	679,617
New Mexico - 2000+	19.9#	2.1	15.9	24.6	87	0.7251	0.5798	0.8961	0.0021	437,101
Seattle (Puget Sound) - 2000+	27.3	1.6	24.3	30.6	296	0.9939	0.8811	1.1175	0.9485	1,084,987
Utah - 2000+	23.3	2.2	19.1	28	111	0.8478	0.6961	1.0231	0.0877	476,974
Atlanta (Metropolitan) - 2000+	25.3	1.8	21.9	29.1	198	0.9215	0.7955	1.062	0.2721	782,792
San Jose-Monterey - 2000+	25.1	2.1	21.2	29.5	146	0.9148	0.7707	1.0783	0.3063	581,437
Los Angeles - 2000+	25.6	1.1	23.6	27.8	565	0.9343	0.8552	1.0191	0.1276	2,203,128
Alaska Natives - 2000+	46.7	14.1	23.3	83.5	11	1.7004	0.8483	3.045	0.1306	23,566
Rural Georgia - 2000+	38.7	11.7	19.3	69.3	11	1.4115	0.7042	2.5276	0.3288	28,390
California excluding SF/SJM/LA - 2000+	24.6#	0.7	23.1	26.1	1,096	0.8951	0.838	0.9554	0.0008	4,460,695
Kentucky - 2000+	34.9#	1.9	31.3	38.8	348	1.2717	1.1377	1.4176	0	996,905
Louisiana - 2000+	37.2#	1.9	33.5	41.2	369	1.3538	1.2149	1.5047	0	992,963
New Jersey - 2000+	28	1.1	25.8	30.3	606	1.021	0.9371	1.1107	0.6407	2,162,333
Greater Georgia - 2000+	31.2#	1.5	28.3	34.4	421	1.138	1.0281	1.2569	0.0128	1,347,723

Age	48	48	48	48	48	48	48	48	48	48
	Rate	SE	Lower CI	Upper CI	Count	Rate Ratio	Ratio Lower CI	Ratio Upper CI	Ratio P-Value	Pop
SEER 18	31.3	0.4	30.5	32.1	6,035					19,286,247
San Francisco-Oakland SMSA - 2000+	27.3#	1.6	24.2	30.7	282	0.8724	0.7715	0.9832	0.0245	1,032,981
Connecticut - 2000+	31.5	1.9	27.9	35.4	279	1.0064	0.8894	1.135	0.9335	885,902
Detroit (Metropolitan) - 2000+	32.9	1.9	29.4	36.8	315	1.052	0.9364	1.1784	0.3948	956,854
Hawaii - 2000+	36.7	3.5	30.2	44.3	110	1.1733	0.9627	1.4168	0.1127	299,613
Iowa - 2000+	32	2.2	27.9	36.5	219	1.0217	0.8887	1.1693	0.774	685,008
New Mexico - 2000+	28.9	2.6	24.1	34.3	128	0.9229	0.7686	1.0996	0.3944	443,212
Seattle (Puget Sound) - 2000+	27.7#	1.6	24.6	31	297	0.8845	0.7846	0.9939	0.0388	1,073,077
Utah - 2000+	25.0#	2.3	20.7	29.9	118	0.7991	0.6603	0.9588	0.0145	471,894
Atlanta (Metropolitan) - 2000+	30.7	2	26.9	34.8	236	0.9799	0.8568	1.1162	0.7925	769,630
San Jose-Monterey - 2000+	31.8	2.4	27.3	36.8	182	1.0162	0.872	1.1778	0.8516	572,358
Los Angeles - 2000+	27.9#	1.1	25.7	30.2	604	0.8925	0.8195	0.9705	0.0073	2,162,804
Alaska Natives - 2000+	69.3#	17.3	39.6	112.6	16	2.2161	1.2659	3.6018	0.0065	23,073
Rural Georgia - 2000+	27.4	9.7	11.8	54	8	0.8761	0.3781	1.7275	0.8773	29,180
California excluding SF/SJM/LA - 2000+	28.2#	0.8	26.7	29.9	1,246	0.9028	0.8487	0.9597	0.0009	4,410,715
Kentucky - 2000+	39.1#	2	35.3	43.2	390	1.2506	1.126	1.3857	0	996,579
Louisiana - 2000+	41.2#	2	37.3	45.4	409	1.3162	1.1878	1.455	0	993,076
New Jersey - 2000+	32.7	1.2	30.3	35.2	701	1.0454	0.9654	1.1306	0.2752	2,142,948
Greater Georgia - 2000+	37.0#	1.7	33.8	40.4	495	1.1829	1.0771	1.2966	0.0005	1,337,343
Age	49	49	49	49	49	49	49	49	49	49
	Rate	SE	Lower CI	Upper CI	Count	Rate Ratio	Ratio Lower CI	Ratio Upper CI	Ratio P-Value	Pop
SEER 18	34.9	0.4	34.1	35.8	6,689					19,163,643
San Francisco-Oakland SMSA - 2000+	33.3	1.8	29.9	37	342	0.9546	0.8538	1.0643	0.4186	1,026,440
Connecticut - 2000+	34.4	2	30.7	38.5	304	0.9866	0.8766	1.107	0.8477	882,754
Detroit (Metropolitan) - 2000+	34.4	1.9	30.8	38.3	327	0.985	0.8788	1.1008	0.8177	951,094
Hawaii - 2000+	42	3.7	35	50	126	1.2022	0.9999	1.434	0.0502	300,260
Iowa - 2000+	32.9	2.2	28.7	37.4	226	0.9414	0.8208	1.0749	0.3907	687,816
New Mexico - 2000+	31.1	2.6	26.1	36.7	138	0.8896	0.7461	1.053	0.1832	444,438
Seattle (Puget Sound) - 2000+	30.5#	1.7	27.3	34	327	0.8731	0.779	0.9758	0.0159	1,072,992
Utah - 2000+	25.2#	2.3	20.9	30.2	118	0.7228	0.5973	0.8671	0.0003	467,739
Atlanta (Metropolitan) - 2000+	32.8	2.1	28.9	37.2	248	0.9401	0.8248	1.0673	0.3561	755,790
San Jose-Monterey - 2000+	32	2.4	27.5	37	181	0.9171	0.7868	1.0631	0.2641	565,456
Los Angeles - 2000+	31.5#	1.2	29.1	33.9	671	0.9012	0.8312	0.9758	0.0099	2,133,069
Alaska Natives - 2000+	70.0#	17.5	40	113.6	16	2.0049	1.1453	3.2582	0.0162	22,864
Rural Georgia - 2000+	67.9#	15.2	41.4	104.8	20	1.9441	1.1867	3.0049	0.0094	29,474
California excluding SF/SJM/LA - 2000+	32.3#	0.9	30.7	34.1	1,418	0.9265	0.8743	0.9813	0.0089	4,384,758
Kentucky - 2000+	47.1#	2.2	42.9	51.5	468	1.3482	1.2249	1.4808	0	994,544
Louisiana - 2000+	47.5#	2.2	43.3	52	472	1.3614	1.2375	1.4949	0	993,252
New Jersey - 2000+	34.3	1.3	31.9	36.9	729	0.9829	0.9093	1.0612	0.676	2,124,823
Greater Georgia - 2000+	42.1#	1.8	38.7	45.7	558	1.2055	1.1038	1.3145	0	1,326,080

Age	50	50	50	50	50	50	50	50	50	50
	Rate	SE	Lower CI	Upper CI	Count	Rate Ratio	Ratio Lower CI	Ratio Upper CI	Ratio P-Value	Pop
SEER 18	51	0.5	50	52.1	9,654					18,918,753
San Francisco-Oakland SMSA - 2000+	51	2.2	46.7	55.5	520	0.9989	0.9128	1.0912	1	1,020,161
Connecticut - 2000+	62.5#	2.7	57.3	68	543	1.2243	1.1208	1.335	0	869,169
Detroit (Metropolitan) - 2000+	49	2.3	44.6	53.7	461	0.96	0.8725	1.0542	0.4062	941,046
Hawaii - 2000+	65.9#	4.7	57.1	75.7	200	1.291	1.1167	1.4852	0.0006	303,584
Iowa - 2000+	55.5	2.8	50.1	61.4	382	1.0885	0.9802	1.2059	0.1125	687,709
New Mexico - 2000+	35.4#	2.8	30	41.3	157	0.6928	0.5879	0.8111	0	444,104
Seattle (Puget Sound) - 2000+	46.4#	2.1	42.4	50.7	494	0.9102	0.8298	0.9964	0.0413	1,063,628
Utah - 2000+	49.7	3.3	43.5	56.6	229	0.9749	0.8514	1.1115	0.735	460,336
Atlanta (Metropolitan) - 2000+	49.9	2.6	44.9	55.2	368	0.9773	0.8783	1.0846	0.6894	737,930
San Jose-Monterey - 2000+	55.3	3.2	49.3	61.9	307	1.0845	0.9648	1.2151	0.1734	554,757
Los Angeles - 2000+	47.6#	1.5	44.7	50.6	997	0.9323	0.8726	0.9952	0.0352	2,095,680
Alaska Natives - 2000+	106.0#	21.6	67.9	157.8	24	2.0781	1.3308	3.094	0.0018	22,632
Rural Georgia - 2000+	68.4	15.3	41.8	105.6	20	1.34	0.8182	2.0708	0.2418	29,248
California excluding SF/SJM/LA - 2000+	44.9#	1	42.9	46.9	1,938	0.879	0.8367	0.923	0	4,320,725
Kentucky - 2000+	61.6#	2.5	56.8	66.7	609	1.2074	1.1106	1.3105	0	988,469
Louisiana - 2000+	59.4#	2.5	54.7	64.5	586	1.1649	1.0698	1.2663	0.0005	985,852
New Jersey - 2000+	49.7	1.5	46.7	52.8	1,037	0.974	0.9127	1.0386	0.431	2,086,372
Greater Georgia - 2000+	59.8#	2.1	55.7	64.2	782	1.1722	1.0884	1.2609	0	1,307,351
Age	51	51	51	51	51	51	51	51	51	51
	Rate	SE	Lower CI	Upper CI	Count	Rate Ratio	Ratio Lower CI	Ratio Upper CI	Ratio P-Value	Pop
SEER 18	51.5	0.5	50.5	52.6	9,639					18,700,852
San Francisco-Oakland SMSA - 2000+	51.7	2.3	47.4	56.4	522	1.0036	0.9173	1.0962	0.9484	1,009,088
Connecticut - 2000+	58.1#	2.6	53.1	63.4	500	1.127	1.0281	1.2332	0.0109	860,737
Detroit (Metropolitan) - 2000+	49.2	2.3	44.8	53.9	460	0.9546	0.8675	1.0483	0.3424	934,893
Hawaii - 2000+	69.6#	4.8	60.5	79.7	209	1.35	1.1715	1.5484	0	300,359
Iowa - 2000+	53.8	2.8	48.5	59.6	368	1.044	0.9382	1.1586	0.4317	683,899
New Mexico - 2000+	46.6	3.2	40.5	53.4	206	0.9042	0.7838	1.0381	0.1593	441,993
Seattle (Puget Sound) - 2000+	42.5#	2	38.6	46.6	450	0.8237	0.7478	0.9055	0	1,059,863
Utah - 2000+	45.6	3.2	39.6	52.3	208	0.8852	0.7679	1.0156	0.0838	455,868
Atlanta (Metropolitan) - 2000+	51.9	2.7	46.8	57.5	374	1.0072	0.9059	1.1169	0.9066	720,436
San Jose-Monterey - 2000+	48	3	42.4	54.2	263	0.9317	0.8211	1.0532	0.2694	547,664
Los Angeles - 2000+	46.2#	1.5	43.3	49.2	950	0.8959	0.8373	0.9578	0.0011	2,057,251
Alaska Natives - 2000+	114.2#	22.8	73.9	168.6	25	2.2162	1.4335	3.2735	0.0006	21,886
Rural Georgia - 2000+	55	13.7	31.4	89.3	16	1.067	0.6097	1.7337	0.8631	29,092
California excluding SF/SJM/LA - 2000+	47.9#	1.1	45.8	50	2,041	0.9292	0.8854	0.9748	0.0025	4,261,477
Kentucky - 2000+	64.4#	2.6	59.4	69.6	632	1.2486	1.1502	1.3534	0	982,021
Louisiana - 2000+	61.8#	2.5	57	66.9	607	1.1989	1.1027	1.3016	0	982,240
New Jersey - 2000+	49.7	1.6	46.7	52.8	1,024	0.9641	0.9031	1.0284	0.2728	2,060,625
Greater Georgia - 2000+	60.7#	2.2	56.5	65.1	784	1.1778	1.0937	1.2669	0	1,291,460

Age	52	52	52	52	52	52	52	52	52	52
	Rate	SE	Lower CI	Upper CI	Count	Rate Ratio	Ratio Lower CI	Ratio Upper CI	Ratio P-Value	Pop
SEER 18	50.6	0.5	49.5	51.6	9,267					18,326,266
San Francisco-Oakland SMSA - 2000+	47	2.2	42.8	51.5	466	0.9294	0.845	1.0201	0.1262	991,576
Connecticut - 2000+	48.5	2.4	43.9	53.5	409	0.9597	0.8671	1.0598	0.4318	842,774
Detroit (Metropolitan) - 2000+	52.2	2.4	47.6	57.1	481	1.032	0.9397	1.1312	0.5129	921,707
Hawaii - 2000+	60.7#	4.5	52.1	70.2	180	1.2001	1.0297	1.3909	0.0199	296,609
Iowa - 2000+	48.4	2.7	43.3	53.9	327	0.9564	0.854	1.0681	0.447	676,116
New Mexico - 2000+	45.4	3.2	39.3	52.2	199	0.8977	0.7761	1.0331	0.1371	438,396
Seattle (Puget Sound) - 2000+	44.8#	2.1	40.9	49.1	467	0.8865	0.8061	0.9729	0.0105	1,041,803
Utah - 2000+	45.7	3.2	39.6	52.4	204	0.903	0.7822	1.0375	0.1558	446,758
Atlanta (Metropolitan) - 2000+	50.3	2.7	45.2	55.8	351	0.9947	0.8916	1.1067	0.95	697,849
San Jose-Monterey - 2000+	48.1	3	42.4	54.3	256	0.9508	0.8365	1.0767	0.4468	532,437
Los Angeles - 2000+	48.8	1.6	45.8	52	978	0.9658	0.9033	1.0317	0.3079	2,002,625
Alaska Natives - 2000+	106.1#	22.6	66.5	160.7	22	2.0986	1.3146	3.1793	0.0025	20,731
Rural Georgia - 2000+	58.8	14.3	34.2	94.1	17	1.1624	0.6768	1.8621	0.6013	28,923
California excluding SF/SJM/LA - 2000+	44.1#	1	42.2	46.2	1,842	0.873	0.83	0.9179	0	4,172,446
Kentucky - 2000+	65.7#	2.6	60.7	71	636	1.3	1.1977	1.4089	0	967,529
Louisiana - 2000+	66.2#	2.6	61.2	71.6	639	1.31	1.2072	1.4196	0	964,607
New Jersey - 2000+	52.1	1.6	49	55.3	1,051	1.0303	0.9657	1.0983	0.3672	2,017,302
Greater Georgia - 2000+	58.6#	2.2	54.5	63	742	1.159	1.074	1.2491	0.0002	1,266,078
Age	53	53	53	53	53	53	53	53	53	53
	Rate	SE	Lower CI	Upper CI	Count	Rate Ratio	Ratio Lower CI	Ratio Upper CI	Ratio P-Value	Pop
SEER 18	53.8	0.5	52.7	54.9	9,721					18,073,808
San Francisco-Oakland SMSA - 2000+	47.0#	2.2	42.8	51.5	460	0.8744	0.7946	0.9603	0.0045	978,083
Connecticut - 2000+	53.6	2.5	48.7	58.8	447	0.9957	0.9036	1.0948	0.9536	834,699
Detroit (Metropolitan) - 2000+	52.8	2.4	48.2	57.8	482	0.9826	0.8948	1.0768	0.7281	912,063
Hawaii - 2000+	60.1	4.5	51.6	69.6	177	1.117	0.9572	1.296	0.1595	294,630
Iowa - 2000+	53.3	2.8	47.9	59.1	357	0.9911	0.8892	1.1016	0.8952	669,745
New Mexico - 2000+	51.3	3.4	44.8	58.4	223	0.953	0.8308	1.0884	0.5017	435,054
Seattle (Puget Sound) - 2000+	45.5#	2.1	41.5	49.8	469	0.8458	0.7693	0.928	0.0003	1,030,958
Utah - 2000+	47	3.3	40.8	53.8	206	0.8734	0.7571	1.0027	0.0549	438,509
Atlanta (Metropolitan) - 2000+	51.1	2.7	45.9	56.8	349	0.9508	0.852	1.058	0.369	682,470
San Jose-Monterey - 2000+	49	3.1	43.2	55.3	257	0.9106	0.8014	1.0307	0.1433	524,748
Los Angeles - 2000+	50.5	1.6	47.4	53.7	988	0.9383	0.878	1.0019	0.0572	1,957,690
Alaska Natives - 2000+	124.4#	24.9	80.5	183.6	25	2.313	1.4961	3.4165	0.0003	20,096
Rural Georgia - 2000+	88.5#	17.4	57.8	129.6	26	1.6451	1.0741	2.412	0.023	29,384
California excluding SF/SJM/LA - 2000+	47.9#	1.1	45.8	50.1	1,966	0.8906	0.848	0.9349	0	4,104,338
Kentucky - 2000+	66.4#	2.6	61.3	71.8	638	1.2345	1.1377	1.3376	0	960,848
Louisiana - 2000+	68.5#	2.7	63.4	74	657	1.2742	1.1756	1.3791	0	958,674
New Jersey - 2000+	56.6	1.7	53.4	60	1,128	1.0527	0.9889	1.1198	0.1073	1,992,215
Greater Georgia - 2000+	69.3#	2.4	64.8	74.1	866	1.2885	1.2006	1.3814	0	1,249,604

Age	54	54	54	54	54	54	54	54	54	54
	Rate	SE	Lower CI	Upper CI	Count	Rate Ratio	Ratio Lower CI	Ratio Upper CI	Ratio P-Value	Pop
SEER 18	58.2	0.6	57.1	59.4	10,245					17,590,040
San Francisco-Oakland SMSA - 2000+	54.6	2.4	50	59.5	520	0.9379	0.8572	1.0244	0.1584	951,891
Connecticut - 2000+	57.8	2.7	52.7	63.3	471	0.993	0.9035	1.0892	0.9055	814,387
Detroit (Metropolitan) - 2000+	55.5	2.5	50.7	60.6	493	0.953	0.8689	1.0432	0.3065	888,225
Hawaii - 2000+	65.8	4.7	56.8	75.8	193	1.1297	0.9746	1.3027	0.105	293,328
Iowa - 2000+	53.7	2.9	48.3	59.7	352	0.9227	0.8274	1.0263	0.1424	654,962
New Mexico - 2000+	49.7#	3.4	43.3	56.9	213	0.8535	0.7417	0.9776	0.0212	428,492
Seattle (Puget Sound) - 2000+	50.7#	2.2	46.4	55.2	510	0.8697	0.7942	0.9507	0.0018	1,006,814
Utah - 2000+	47.9#	3.4	41.5	54.9	204	0.8217	0.7118	0.9439	0.0048	426,272
Atlanta (Metropolitan) - 2000+	55.9	2.9	50.3	61.9	366	0.9598	0.8624	1.0654	0.4583	654,716
San Jose-Monterey - 2000+	51.3#	3.2	45.3	58	261	0.8814	0.7765	0.9966	0.0438	508,446
Los Angeles - 2000+	55.9	1.7	52.6	59.3	1,061	0.9593	0.8997	1.022	0.2018	1,899,032
Alaska Natives - 2000+	114.8#	24.5	72	173.9	22	1.9718	1.2352	2.987	0.0054	19,156
Rural Georgia - 2000+	69	15.4	42.2	106.6	20	1.1855	0.7238	1.8319	0.5069	28,965
California excluding SF/SJM/LA - 2000+	52.9#	1.2	50.7	55.2	2,112	0.9089	0.867	0.9526	0.0001	3,989,416
Kentucky - 2000+	78.1#	2.9	72.6	84	733	1.3417	1.2431	1.4462	0	938,014
Louisiana - 2000+	75.7#	2.8	70.2	81.5	708	1.3001	1.2031	1.4032	0	934,988
New Jersey - 2000+	56.4	1.7	53.1	59.8	1,091	0.9676	0.9083	1.0301	0.309	1,935,821
Greater Georgia - 2000+	75.2#	2.5	70.4	80.2	915	1.2908	1.205	1.3812	0	1,217,115
Age	55	55	55	55	55	55	55	55	55	55
	Rate	SE	Lower CI	Upper CI	Count	Rate Ratio	Ratio Lower CI	Ratio Upper CI	Ratio P-Value	Pop
SEER 18	62.1	0.6	60.9	63.3	10,538					16,965,058
San Francisco-Oakland SMSA - 2000+	54.1#	2.4	49.5	59.1	501	0.871	0.7948	0.9528	0.0022	925,989
Connecticut - 2000+	59.9	2.8	54.6	65.5	470	0.9636	0.8767	1.057	0.4465	785,260
Detroit (Metropolitan) - 2000+	68.0#	2.8	62.6	73.7	582	1.0944	1.0051	1.1898	0.0379	856,126
Hawaii - 2000+	73.4#	5.1	63.8	84.1	210	1.1822	1.0263	1.3553	0.0206	285,976
Iowa - 2000+	62.7	3.1	56.7	69.2	398	1.0101	0.9116	1.1166	0.8575	634,307
New Mexico - 2000+	51.3#	3.5	44.7	58.7	213	0.8263	0.718	0.9464	0.0051	415,010
Seattle (Puget Sound) - 2000+	52.6#	2.3	48.1	57.3	511	0.8462	0.7728	0.9249	0.0002	972,124
Utah - 2000+	45.7#	3.3	39.4	52.8	187	0.7364	0.6339	0.8511	0	408,787
Atlanta (Metropolitan) - 2000+	67.4	3.3	61.1	74.2	421	1.0853	0.9822	1.1965	0.1076	624,500
San Jose-Monterey - 2000+	55.7	3.4	49.3	62.7	271	0.8966	0.7918	1.0116	0.0776	486,572
Los Angeles - 2000+	56.3#	1.8	52.9	59.9	1,029	0.907	0.8499	0.967	0.0026	1,826,536
Alaska Natives - 2000+	127.6#	26	81.8	189.9	24	2.0543	1.3156	3.0583	0.0021	18,808
Rural Georgia - 2000+	88.1	17.6	57	130	25	1.4178	0.9171	2.0941	0.1143	28,387
California excluding SF/SJM/LA - 2000+	54.9#	1.2	52.6	57.3	2,108	0.8839	0.8432	0.9264	0	3,839,206
Kentucky - 2000+	85.7#	3.1	79.8	91.9	781	1.3795	1.2812	1.4837	0	911,424
Louisiana - 2000+	77.4#	2.9	71.8	83.4	700	1.2466	1.1532	1.3459	0	903,995
New Jersey - 2000+	66.0#	1.9	62.3	69.8	1,230	1.062	1.0003	1.1268	0.0487	1,864,489
Greater Georgia - 2000+	74.5#	2.5	69.6	79.6	877	1.199	1.1179	1.2846	0	1,177,562

Age	56	56	56	56	56	56	56	56	56	56
	Rate	SE	Lower CI	Upper CI	Count	Rate Ratio	Ratio Lower CI	Ratio Upper CI	Ratio P-Value	Pop
SEER 18	66.4	0.6	65.2	67.7	10,903					16,416,007
San Francisco-Oakland SMSA - 2000+	58.5#	2.6	53.6	63.7	523	0.8805	0.805	0.9613	0.0041	894,315
Connecticut - 2000+	61.8	2.8	56.3	67.6	470	0.9299	0.846	1.0199	0.1258	761,032
Detroit (Metropolitan) - 2000+	66.7	2.8	61.3	72.5	553	1.0048	0.9208	1.0945	0.925	828,656
Hawaii - 2000+	85.6#	5.5	75.1	97.1	239	1.2885	1.1288	1.4648	0.0002	279,268
Iowa - 2000+	67.1	3.3	60.8	73.9	413	1.0103	0.9136	1.1147	0.8516	615,480
New Mexico - 2000+	60.5	3.9	53.2	68.6	246	0.9112	0.7998	1.034	0.1549	406,482
Seattle (Puget Sound) - 2000+	54.2#	2.4	49.6	59.1	512	0.8158	0.7452	0.8915	0	944,900
Utah - 2000+	55.6#	3.7	48.5	63.5	221	0.8377	0.7299	0.9571	0.0083	397,203
Atlanta (Metropolitan) - 2000+	64.7	3.3	58.4	71.5	385	0.9737	0.8773	1.0779	0.6293	595,341
San Jose-Monterey - 2000+	49.1#	3.2	43	55.9	230	0.7394	0.646	0.8426	0	468,349
Los Angeles - 2000+	64	1.9	60.3	67.9	1,124	0.9637	0.9055	1.0248	0.2433	1,756,099
Alaska Natives - 2000+	186.3#	32.4	128.2	261.6	33	2.8046	1.9296	3.9411	0	17,716
Rural Georgia - 2000+	91.3	17.9	59.6	133.7	26	1.3743	0.8974	2.0148	0.1413	28,484
California excluding SF/SJM/LA - 2000+	59.6#	1.3	57.2	62.2	2,212	0.8981	0.8576	0.9402	0	3,708,422
Kentucky - 2000+	87.5#	3.1	81.4	93.8	777	1.3169	1.2229	1.4165	0	888,343
Louisiana - 2000+	82.0#	3.1	76.2	88.2	721	1.2351	1.1439	1.332	0	878,900
New Jersey - 2000+	71.5#	2	67.7	75.5	1,287	1.0769	1.0156	1.141	0.0132	1,799,448
Greater Georgia - 2000+	81.1#	2.7	76	86.5	931	1.2215	1.1412	1.3062	0	1,147,569
Age	57	57	57	57	57	57	57	57	57	57
	Rate	SE	Lower CI	Upper CI	Count	Rate Ratio	Ratio Lower CI	Ratio Upper CI	Ratio P-Value	Pop
SEER 18	70.8	0.7	69.5	72.2	11,238					15,862,397
San Francisco-Oakland SMSA - 2000+	65.6	2.8	60.3	71.3	567	0.9262	0.8498	1.0079	0.0761	864,064
Connecticut - 2000+	72.8	3.1	66.8	79.2	536	1.0274	0.9404	1.1205	0.5525	736,369
Detroit (Metropolitan) - 2000+	72.2	3	66.4	78.3	578	1.0189	0.9356	1.1078	0.6727	800,723
Hawaii - 2000+	81.5#	5.5	71.2	93	223	1.1508	1.0034	1.314	0.0446	273,510
Iowa - 2000+	71.4	3.5	64.8	78.5	427	1.0082	0.9132	1.1105	0.8828	597,836
New Mexico - 2000+	60.7#	3.9	53.3	68.9	241	0.857	0.7512	0.9737	0.0169	396,943
Seattle (Puget Sound) - 2000+	55.9#	2.5	51.1	60.9	511	0.7884	0.7201	0.8616	0	914,849
Utah - 2000+	55.4#	3.8	48.2	63.3	213	0.7813	0.6791	0.8948	0.0002	384,792
Atlanta (Metropolitan) - 2000+	70	3.5	63.3	77.2	398	0.988	0.8918	1.092	0.8383	568,606
San Jose-Monterey - 2000+	56.1#	3.5	49.3	63.4	252	0.7912	0.6955	0.8964	0.0002	449,589
Los Angeles - 2000+	67.8	2	63.9	71.9	1,140	0.9571	0.8998	1.0174	0.162	1,681,178
Alaska Natives - 2000+	77.6	21.5	41.3	132.7	13	1.0957	0.5832	1.8744	0.8174	16,747
Rural Georgia - 2000+	82.1	17.1	52.1	123.3	23	1.1594	0.7347	1.7406	0.5345	28,000
California excluding SF/SJM/LA - 2000+	64.0#	1.3	61.4	66.7	2,293	0.9038	0.8638	0.9454	0	3,580,912
Kentucky - 2000+	91.8#	3.3	85.5	98.4	789	1.2952	1.2035	1.3923	0	859,857
Louisiana - 2000+	99.0#	3.4	92.4	105.9	840	1.3975	1.3014	1.4992	0	848,399
New Jersey - 2000+	72.4	2	68.4	76.5	1,262	1.0213	0.9628	1.0825	0.4868	1,744,240
Greater Georgia - 2000+	83.5#	2.7	78.3	89.1	932	1.179	1.1016	1.2606	0	1,115,783

Age	58	58	58	58	58	58	58	58	58	58
	Rate	SE	Lower CI	Upper CI	Count	Rate Ratio	Ratio Lower CI	Ratio Upper CI	Ratio P-Value	Pop
SEER 18	75.6	0.7	74.2	77	11,525					15,244,402
San Francisco-Oakland SMSA - 2000+	65.7#	2.8	60.3	71.4	544	0.8687	0.7956	0.9468	0.0012	828,339
Connecticut - 2000+	73.4	3.2	67.3	80	520	0.9713	0.8879	1.0606	0.5333	708,127
Detroit (Metropolitan) - 2000+	80.9	3.2	74.7	87.5	622	1.0701	0.9856	1.1601	0.1064	768,842
Hawaii - 2000+	83.4	5.6	72.8	95.2	221	1.1037	0.9618	1.2609	0.1593	264,852
Iowa - 2000+	72.6	3.5	65.8	79.9	419	0.9602	0.8689	1.0586	0.4294	577,212
New Mexico - 2000+	70	4.3	61.8	78.8	269	0.9254	0.8169	1.0444	0.2175	384,510
Seattle (Puget Sound) - 2000+	59.6#	2.6	54.6	64.9	524	0.788	0.7206	0.8602	0	879,536
Utah - 2000+	51.0#	3.7	43.9	58.8	188	0.6741	0.5805	0.7786	0	368,920
Atlanta (Metropolitan) - 2000+	75.8	3.7	68.6	83.5	409	1.0023	0.906	1.1063	0.9775	539,755
San Jose-Monterey - 2000+	62.8#	3.8	55.5	70.8	269	0.831	0.7336	0.9378	0.0023	428,192
Los Angeles - 2000+	74.1	2.1	70	78.5	1,195	0.9806	0.9232	1.0409	0.5316	1,611,851
Alaska Natives - 2000+	159.7#	31.9	103.3	235.7	25	2.1123	1.3664	3.1198	0.0011	15,655
Rural Georgia - 2000+	73.6	16.4	44.9	113.6	20	0.9729	0.5941	1.5034	1	27,190
California excluding SF/SJM/LA - 2000+	67.7#	1.4	65	70.6	2,325	0.8961	0.8567	0.9371	0	3,431,765
Kentucky - 2000+	92.8#	3.3	86.4	99.6	777	1.2281	1.1406	1.3208	0	836,869
Louisiana - 2000+	103.2#	3.6	96.4	110.4	844	1.3655	1.2718	1.4645	0	817,573
New Jersey - 2000+	80.3#	2.2	76.1	84.7	1,345	1.0625	1.0034	1.1243	0.0378	1,674,412
Greater Georgia - 2000+	93.4#	2.9	87.7	99.3	1,009	1.2349	1.1568	1.317	0	1,080,802

Age	59	59	59	59	59	59	59	59	59	59
	Rate	SE	Lower CI	Upper CI	Count	Rate Ratio	Ratio Lower CI	Ratio Upper CI	Ratio P-Value	Pop
SEER 18	82.3	0.8	80.8	83.8	11,991					14,571,936
San Francisco-Oakland SMSA - 2000+	74.4#	3.1	68.5	80.6	587	0.9039	0.8306	0.982	0.0164	789,205
Connecticut - 2000+	76.1	3.4	69.7	83	514	0.9254	0.8455	1.0109	0.0866	675,014
Detroit (Metropolitan) - 2000+	86.9	3.4	80.2	93.9	635	1.0557	0.9732	1.1436	0.1912	730,934
Hawaii - 2000+	99.7#	6.2	87.8	112.7	255	1.2114	1.0659	1.3715	0.0035	255,803
Iowa - 2000+	79.4	3.8	72.2	87.2	442	0.9651	0.8757	1.0613	0.4803	556,554
New Mexico - 2000+	67.0#	4.3	58.9	75.9	248	0.8139	0.7149	0.923	0.001	370,278
Seattle (Puget Sound) - 2000+	69.4#	2.9	63.8	75.2	582	0.843	0.7744	0.9162	0	839,016
Utah - 2000+	64.5#	4.3	56.4	73.4	227	0.7837	0.6842	0.8937	0.0002	352,001
Atlanta (Metropolitan) - 2000+	82.1	4	74.4	90.4	417	0.9978	0.9028	1.1002	0.9901	507,892
San Jose-Monterey - 2000+	76.4	4.3	68.2	85.4	313	0.9288	0.8276	1.0392	0.2049	409,519
Los Angeles - 2000+	81.1	2.3	76.7	85.7	1,244	0.9857	0.9291	1.0451	0.6415	1,533,690
Alaska Natives - 2000+	128.1	29.4	77.2	200.1	19	1.5573	0.9372	2.4329	0.0858	14,827
Rural Georgia - 2000+	82.1	17.5	51.4	124.2	22	0.9972	0.6247	1.5106	1	26,809
California excluding SF/SJM/LA - 2000+	73.8#	1.5	70.9	76.8	2,421	0.8966	0.858	0.9367	0	3,281,283
Kentucky - 2000+	105.4#	3.6	98.4	112.7	849	1.281	1.1935	1.3735	0	805,425
Louisiana - 2000+	103.5#	3.6	96.5	110.8	816	1.2576	1.1701	1.3502	0	788,505
New Jersey - 2000+	87.6#	2.3	83.1	92.3	1,400	1.0647	1.0067	1.1254	0.0284	1,597,920
Greater Georgia - 2000+	96.4#	3	90.5	102.6	1,000	1.1716	1.0973	1.2498	0	1,037,261

Age	60	60	60	60	60	60	60	60	60	60
	Rate	SE	Lower CI	Upper CI	Count	Rate Ratio	Ratio Lower CI	Ratio Upper CI	Ratio P-Value	Pop
SEER 18	89.8	0.8	88.2	91.4	12,449					13,865,681
San Francisco-Oakland SMSA - 2000+	76.6#	3.2	70.5	83.2	577	0.8536	0.7839	0.928	0.0002	752,858
Connecticut - 2000+	86.5	3.7	79.4	94	554	0.9629	0.8827	1.0485	0.3964	640,832
Detroit (Metropolitan) - 2000+	91.3	3.6	84.3	98.7	631	1.0168	0.9372	1.1016	0.6935	691,165
Hawaii - 2000+	103.1#	6.5	90.8	116.6	253	1.1484	1.01	1.3007	0.0348	245,369
Iowa - 2000+	88.7	4.1	80.8	97	473	0.9875	0.899	1.0826	0.8115	533,497
New Mexico - 2000+	83.7	4.8	74.4	93.7	298	0.9319	0.828	1.0454	0.2386	356,163
Seattle (Puget Sound) - 2000+	74.6#	3.1	68.7	80.8	593	0.8308	0.7638	0.9022	0	794,976
Utah - 2000+	68.5#	4.5	59.9	78	229	0.7628	0.6664	0.8693	0	334,388
Atlanta (Metropolitan) - 2000+	86.4	4.3	78.3	95.2	411	0.9625	0.8703	1.0619	0.4624	475,622
San Jose-Monterey - 2000+	69.7#	4.2	61.6	78.5	269	0.7763	0.6854	0.876	0	385,947
Los Angeles - 2000+	89.1	2.5	84.3	94.1	1,293	0.9921	0.9362	1.0507	0.8011	1,451,557
Alaska Natives - 2000+	236.2#	41.1	162.6	331.6	33	2.6303	1.8097	3.6959	0	13,974
Rural Georgia - 2000+	129.6	22.2	89.8	181.1	34	1.4436	0.9993	2.0183	0.0505	26,233
California excluding SF/SJM/LA - 2000+	79.8#	1.6	76.7	83	2,491	0.8885	0.8508	0.9277	0	3,122,541
Kentucky - 2000+	122.6#	4	114.9	130.6	948	1.3654	1.2768	1.4587	0	773,336
Louisiana - 2000+	114.8#	3.9	107.2	122.7	862	1.2784	1.1917	1.3698	0	751,039
New Jersey - 2000+	94.7	2.5	89.9	99.7	1,441	1.0545	0.9979	1.1137	0.0595	1,521,987
Greater Georgia - 2000+	106.5#	3.3	100.2	113.1	1,059	1.1864	1.1132	1.2633	0	994,197

	Rates are per 100,000 and age-adjusted to the 2000 US Std Population (single ages to 84 - Census P25-1130) standard.
	Confidence intervals are 95% for rates and ratios.
#	The rate ratio indicates that the rate is significantly different than the rate for SEER 18 (p<0.05).
	Warning: Use caution when interpreting ratios and related statistics as the ratio variable contains overlapping groupings.
SEER	The Surveillance, Epidemiology, and End Results (SEER) Program
~	Statistic could not be calculated.
