# Supplementary material for: Geographic and intra‐racial disparities in early‐onset colorectal cancer in the SEER 18 registries of the United States
Source: Cancer Med. 2020 Oct 22;9(23):9150–9. doi: 10.1002/cam4.3488 (PMC7724480; doi:10.1002/cam4.3488)
Supplement: Supplementary file 7 — Supplementary Material [file CAM4-9-9150-s007.pdf]

Supplemental Spreadsheet 3. SEER 18 Registries Incidence Rates Stratified by Race in One Year Age Increments										
	Age	30	31	32	33	34	35	36	37	38
		Rate	Rate	Rate	Rate	Rate	Rate	Rate	Rate	Rate
White	SEER 18	2.8	3.4	3.9	5.1	5.2	6.1	6.7	7.3	8.9
	San Francisco-Oakland SMSA - 2000+	2.3	1.8#	3.6	4.2	5.1	4.2#	7.2	6.6	9.8
	Connecticut - 2000+	2.9	2.9	3	4.9	5.8	7.2	6	7.2	8.4
	Detroit (Metropolitan) - 2000+	3.6	3.6	3.4	5.9	6.9	5.8	5.4	5.8	8.7
	Hawaii - 2000+	1.2	5.3	0	5.4	5.6	12.3	2.8	8.4	14
	Iowa - 2000+	2.2	2.7	3.6	3.6	5.6	7.1	4.9	8.6	8.3
	New Mexico - 2000+	2.7	3.9	3.7	4.6	6.5	4	5.2	6.9	6.5
	Seattle (Puget Sound) - 2000+	2.3	3.6	2.8	5.2	4.4	5.8	7.4	6	8.7
	Utah - 2000+	3.5	4.1	5.2	4.5	3.7	5.3	8.3	5.9	8.9
	Atlanta (Metropolitan) - 2000+	0.9#	4.3	3.6	5.4	4.3	5.4	5.4	8.8	8.3
	San Jose-Monterey - 2000+	2.5	2.8	4.8	6.3	3.8	5.3	4.8	7.8	4.5#
	Los Angeles - 2000+	2.2	2.9	2.8#	4.6	4.0#	5.5	5.5	5.7#	7.7
	Alaska Natives - 2000+	~	~	~	~	~	~	~	~	~
	Rural Georgia - 2000+	0	0	0	15.6	0	0	7.3	14.5	14
	California excluding SF/SJM/LA - 2000+	2.9	3	3.9	4.8	5.1	5.9	6.9	6.8	8.2
	Kentucky - 2000+	4.4#	4.3	6.7#	7.1#	7.0#	8.2#	9.1#	9	12.2#
	Louisiana - 2000+	2.8	4.7	4.4	6.3	7.5#	8.7#	7.9	9.5	10.7
	New Jersey - 2000+	3.2	4.1	3.8	5.2	5.1	6.5	6.4	8.5	10.3
	Greater Georgia - 2000+	3.3	4	4.5	5.9	5.6	6.3	8.1	9	10.5
Black	SEER 18	2.6	3.1	4.1	4.5	5.2	5.2	7.4	8.1	9.7
	San Francisco-Oakland SMSA - 2000+	3.1	3.2	3.2	6.4	2.1	2.1	2.1	15	8.4
	Connecticut - 2000+	1.1	4.4	4.4	6.5	3.3	2.1	9.7	3.2	10.8
	Detroit (Metropolitan) - 2000+	4.2	2.8	2.7	3.2	6.4	5	5.8	8.5	11.6
	Hawaii - 2000+	0	9.2	0	9.7	10.1	20.9	0	0	0
	Iowa - 2000+	0	0	0	4.8	0	4.9	0	20.3	5.4
	New Mexico - 2000+	0	0	0	0	9.1	0	0	9.1	19
	Seattle (Puget Sound) - 2000+	0	6.4	4.8	1.6	8.2	3.2	8.3	1.7	3.4
	Utah - 2000+	0	0	0	0	0	0	12.9	13.2	0
	Atlanta (Metropolitan) - 2000+	2.9	2.9	3.2	4.4	5.8	5.7	8.2	8.2	10.2
	San Jose-Monterey - 2000+	0	0	0	0	5	9.8	5.1	15.3	5.1
	Los Angeles - 2000+	3.6	2.8	3.2	2.8	4.6	4.1	7.2	4.9	8.9
	Alaska Natives - 2000+	~	~	~	~	~	~	~	~	~
	Rural Georgia - 2000+	9.7	0	10.1	0	9.5	0	9.6	0	0
	California excluding SF/SJM/LA - 2000+	1.8	2.6	3.7	4.5	1.9#	3	6.9	7.9	8.3
	Kentucky - 2000+	5	1.3	3.9	7.8	7.8	4	7.9	8	5.4
	Louisiana - 2000+	2.9	3.6	5.9	3.3	4.4	8.3	7.5	9	14.9#
	New Jersey - 2000+	1.3	3.4	3	5.3	6.9	5.2	8.3	8.9	7.6
	Greater Georgia - 2000+	2.4	3.4	6.6	6	6.3	6.9	8.3	8	9.7

	Age	39	40	41	42	43	44	45	46
		Rate	Rate	Rate	Rate	Rate	Rate	Rate	Rate
White	SEER 18	10.1	11.5	13.1	14	16.5	18.4	21.2	24.5
	San Francisco-Oakland SMSA - 2000+	8.4	10.6	11	13.4	16.5	20.3	19.4	22.2
	Connecticut - 2000+	12.7	12.4	11.2	14.2	17.4	19.8	21.7	24.9
	Detroit (Metropolitan) - 2000+	10.5	13.1	10.9	16.5	14.4	15.7	20.4	23.3
	Hawaii - 2000+	9.5	11.8	14.9	19.1	19.9	19.7	23.1	21
	Iowa - 2000+	11.1	15.0#	14.6	15.3	19.6	21.2	20.4	26.6
	New Mexico - 2000+	7.2	7.1#	12	10.5	13.3	17.1	23.1	23.8
	Seattle (Puget Sound) - 2000+	8.6	9.5	10.4#	13.9	13.8	17.9	19.8	18.9#
	Utah - 2000+	11.1	10.8	12.4	13	14.7	14.1#	19.3	23.3
	Atlanta (Metropolitan) - 2000+	7.1#	13.8	13.5	17.1	15.2	15.6	19.7	22.9
	San Jose-Monterey - 2000+	10.2	8.6	13.5	14.7	16.4	18.9	18.1	21.6
	Los Angeles - 2000+	9.1	9.2#	12.6	11.0#	13.7#	14.6#	18.7#	22.4
	Alaska Natives - 2000+	~	~	~	~	~	~	~	~
	Rural Georgia - 2000+	0	6.8	19.9	6.5	6.4	12.5	37.8	50.3
	California excluding SF/SJM/LA - 2000+	9	11.1	12.1	12.6#	15.0#	16.8#	19.1#	22.6#
	Kentucky - 2000+	13.0#	15.4#	17.1#	19.6#	23.3#	25.4#	30.0#	31.6#
	Louisiana - 2000+	14.9#	12.4	14.9	15.1	20.3#	19.9	22.9	30.8#
	New Jersey - 2000+	10.3	11.6	13.7	14.2	18.1	20.1	22.9	26.7
	Greater Georgia - 2000+	12.4#	13.3	18.4#	16.7#	18.8	22.2#	25.6#	28.9#
Black	SEER 18	11.3	14.9	14.1	16.3	20.6	21.9	26.7	31.1
	San Francisco-Oakland SMSA - 2000+	9.4	12	11.3	9.2	17.1	16.3	15.9#	33.7
	Connecticut - 2000+	13	16.1	14.9	12.8	24.5	11.9#	23.2	24.9
	Detroit (Metropolitan) - 2000+	8.1	14.2	12.7	19.6	21.3	22.1	25	30
	Hawaii - 2000+	12.3	12.3	25.3	14.7	30.2	0	69.3	18.1
	Iowa - 2000+	27	10.9	0	5.8	17.2	47.6	17.8	24.7
	New Mexico - 2000+	18.4	9.5	0	9.4	0	9.5	9.7	39.1
	Seattle (Puget Sound) - 2000+	8.5	12	13.7	8.7	14	17.8	20.1	33.6
	Utah - 2000+	0	0	29	15.2	0	52	35.2	17.1
	Atlanta (Metropolitan) - 2000+	10.3	16.4	14.9	14.7	23.5	24.7	29.1	34.9
	San Jose-Monterey - 2000+	15.6	9.8	5	24.9	29.6	4.9	15.9	24.6
	Los Angeles - 2000+	13.7	13	11.2	16.6	20.7	22.2	24.8	31.9
	Alaska Natives - 2000+	~	~	~	~	~	~	~	~
	Rural Georgia - 2000+	27.4	36	8.6	26	17.1	66.3#	33	49.2
	California excluding SF/SJM/LA - 2000+	8.2	9.7#	9.3#	13.9	16.4	15.7#	19.1#	26.8
	Kentucky - 2000+	14.9	18.5	18.6	18.5	22.2	22.3	33.3	30.3
	Louisiana - 2000+	13.2	20.7#	18.3	18.5	19.9	26.2	31	30.9
	New Jersey - 2000+	8.8	15.3	16.1	15.6	19.8	19.5	24.7	26.6
	Greater Georgia - 2000+	14	14.2	15.5	19.8	23.2	25.6	34.7#	36.1

	Age	47	48	49	50	51	52	53	54
		Rate	Rate	Rate	Rate	Rate	Rate	Rate	Rate
White	SEER 18	26.6	30.5	34	49.7	49.6	48.8	51.2	56.2
	San Francisco-Oakland SMSA - 2000+	23.2	26.1#	31.8	50.2	49.9	45.8	44.7#	51.7
	Connecticut - 2000+	26.1	31.3	33.9	61.7#	55.2#	47.6	50.4	55.1
	Detroit (Metropolitan) - 2000+	28.1	31.4	31.2	45.3	42.9#	47.7	49.5	50.6
	Hawaii - 2000+	21.1	43.2	44.3	62.5	72.4#	49.1	54.8	64.1
	Iowa - 2000+	29	31.2	32.8	56.0#	53.9	48	51.4	53.8
	New Mexico - 2000+	18.0#	30.8	28.5	36.6#	47.8	44.8	52.5	49.5
	Seattle (Puget Sound) - 2000+	26.4	26.6#	29.5#	45.9	42.3#	41.7#	43.5#	50.7#
	Utah - 2000+	23.7	25.0#	26.1#	50.7	47.1	46.3	46.7	48.2#
	Atlanta (Metropolitan) - 2000+	25.2	30.2	31.5	44.4	45.7	43.5	45.2	51.1
	San Jose-Monterey - 2000+	24.5	30.7	32.9	54.3	48.8	50.4	49.2	53
	Los Angeles - 2000+	24.1	25.9#	29.7#	45.8#	44.8#	45.7	46.9#	52.2
	Alaska Natives - 2000+	~	~	~	~	~	~	~	~
	Rural Georgia - 2000+	18.7	24	47.5	29.3	35	40.8	72.9	50.8
	California excluding SF/SJM/LA - 2000+	24.4#	28.3#	32.7	43.9#	46.7#	44.5#	47.1#	52.0#
	Kentucky - 2000+	35.7#	38.6#	45.8#	61.9#	63.0#	65.4#	65.4#	77.1#
	Louisiana - 2000+	35.6#	40.3#	43.2#	54.7	56.8#	59.6#	61.0#	69.3#
	New Jersey - 2000+	27.7	32.9	35	50.1	49.3	52.1	54.7	57.4
	Greater Georgia - 2000+	30.1#	34.8#	40.9#	58.9#	56.5#	56.3#	63.2#	70.5#
Black	SEER 18	32.7	37.4	41.9	61.7	65.5	64.9	73.9	76.6
	San Francisco-Oakland SMSA - 2000+	31.5	33.1	40.2	64.1	52.3	58.2	57.4	76.6
	Connecticut - 2000+	28.7	22.6#	38.3	64.7	79.3	59.8	82.9	76.7
	Detroit (Metropolitan) - 2000+	32.6	40.3	46.2	62.8	71.7	67.2	66.9	74.2
	Hawaii - 2000+	0	54.8	20.2	38.8	21.5	22.7	0	73.5
	Iowa - 2000+	51	58.3	45.6	39.5	56.2	86.7	90.9	40.4
	New Mexico - 2000+	49.2	28.5	10	10.2#	31.8	51.1	55	33.7
	Seattle (Puget Sound) - 2000+	39.5	39.3	31.8	53.4	48.8	61.5	74.7	59.1
	Utah - 2000+	18.1	38.3	0	39.9	40.6	45.8	167.2	71.9
	Atlanta (Metropolitan) - 2000+	26.3	32.5	35.5	59.7	62.8	64.7	65.3	64.7
	San Jose-Monterey - 2000+	16.1	53	33	72.7	39.4	30.5	90.9	77
	Los Angeles - 2000+	35.9	27.9#	34.8	62	55.5	70.6	65.1	81.2
	Alaska Natives - 2000+	~	~	~	~	~	~	~	~
	Rural Georgia - 2000+	66.3	32.8	97.5#	126.0#	85.1	86.3	114.7	99.6
	California excluding SF/SJM/LA - 2000+	27.5	33.9	32.6#	56.9	67.4	44.9#	62.2	66.6
	Kentucky - 2000+	27.9	47.5	55.3	53.9	76.8	71.9	71	90.4
	Louisiana - 2000+	41.5#	44.5	58.9#	71	75.2	82.4#	88.2#	92.0#
	New Jersey - 2000+	30.4	37	35.2	56	52.7#	57.7	72.9	61.2#
	Greater Georgia - 2000+	34.7	43.6	47	64.7	75	67.8	88.2#	92.0#

	Age	55	56	57	58	59	60
		Rate	Rate	Rate	Rate	Rate	Rate
White	SEER 18	60.2	63.5	68.1	73.1	79.4	87.6
	San Francisco-Oakland SMSA - 2000+	52.3#	53.5#	60.2#	62.0#	69.0#	74.9#
	Connecticut - 2000+	58.3	60.5	72.7	72.3	73.4	84.6
	Detroit (Metropolitan) - 2000+	62.6	59.8	65.9	70.1	78.6	84.5
	Hawaii - 2000+	75.5	82.0#	80.2	70.1	87	93.1
	Iowa - 2000+	63.1	67.6	70.6	71.4	79.8	87.6
	New Mexico - 2000+	53.6	60.6	61.2	71.2	67.6#	85.7
	Seattle (Puget Sound) - 2000+	51.5#	52.8#	55.5#	59.0#	67.8#	73.9#
	Utah - 2000+	45.1#	56.7	55.6#	51.8#	64.2#	70.2#
	Atlanta (Metropolitan) - 2000+	57	56.7	62.2	67.2	70.1	77.4
	San Jose-Monterey - 2000+	54.6	48.3#	52.8#	63.6	77.9	65.1#
	Los Angeles - 2000+	55.8#	59.8	62.0#	72.4	78.2	86
	Alaska Natives - 2000+	~	~	~	~	~	~
	Rural Georgia - 2000+	90	50.3	61.4	63	56.6	120.2
	California excluding SF/SJM/LA - 2000+	54.5#	58.6#	63.5#	67.4#	73.3#	80.7#
	Kentucky - 2000+	84.3#	85.2#	90.7#	91.3#	103.9#	121.4#
	Louisiana - 2000+	70.9#	73.6#	91.6#	92.3#	96.5#	104.8#
	New Jersey - 2000+	65.0#	71.6#	71.6	81.9#	88.5#	95.1#
	Greater Georgia - 2000+	71.6#	76.1#	78.9#	90.1#	90.3#	102.1#
Black	SEER 18	81.6	89.3	95.4	102.2	111.6	116.9
	San Francisco-Oakland SMSA - 2000+	53.2#	63.1#	89.2	82.1	110.6	108.3
	Connecticut - 2000+	70.6	69.1	82.5	98.6	101.8	82.3#
	Detroit (Metropolitan) - 2000+	84.4	94.2	98.9	115.3	117.3	116.2
	Hawaii - 2000+	138.5	89.4	64.1	31.3	74.7	39.7
	Iowa - 2000+	76.7	36.4	77.7	140.2	66.3	144.1
	New Mexico - 2000+	24.4	38.4	51.3	81.5	29.3#	32.0#
	Seattle (Puget Sound) - 2000+	76.3	75.8	85.8	70.1	105.6	60.8#
	Utah - 2000+	25.8	0	0	68.2	73.6	0
	Atlanta (Metropolitan) - 2000+	86.8	79.7	85.9	94.6	106.5	111
	San Jose-Monterey - 2000+	89.7	49.7	99.8	75.1	107.1	96
	Los Angeles - 2000+	72.4	97.1	95	96	104.6	122.8
	Alaska Natives - 2000+	~	~	~	~	~	~
	Rural Georgia - 2000+	76.6	163.5#	121.2	94	133.4	151.1
	California excluding SF/SJM/LA - 2000+	66.1#	75	75.5#	88.5	101.9	97.3#
	Kentucky - 2000+	106.9#	112.5	105.2	115.6	131.7	144.5
	Louisiana - 2000+	95.7#	106.4#	121.9#	133.8#	123.9	145.5#
	New Jersey - 2000+	82.8	84	89.3	85.5#	101.9	105.8
	Greater Georgia - 2000+	86.7	100.1	100.4	107.2	123.1	128.9

	Rates are per 100,000 and age-adjusted to the 2000 US Std Population (single ages to 84 - Census P25-1130) standard.
	Confidence intervals are 95% for rates and ratios.
#	The rate ratio indicates that the rate is significantly different than the rate for SEER 18 ($p<0.05$).
	Warning: Use caution when interpreting ratios and related statistics as the ratio variable contains overlapping groupings.
SEER	The Surveillance, Epidemiology, and End Results (SEER) Program
~	Statistic could not be calculated.
