# Supplementary material for: Geographic and intra‐racial disparities in early‐onset colorectal cancer in the SEER 18 registries of the United States
Source: Cancer Med. 2020 Oct 22;9(23):9150–9. doi: 10.1002/cam4.3488 (PMC7724480; doi:10.1002/cam4.3488)
Supplement: Supplementary file 8 — Supplementary Material [file CAM4-9-9150-s008.docx]

**Supplemental Methods File: Colorectal Cancer Classification and Histologic Inclusion Criteria**

International Classification of Diseases 0-3 codes: C18.0–C18.9, C26.0 for colon cancer and C19.9/C20.9 for rectal cancer and histology codes 8140-8147, 8210-8213, 8220-8221, 8260-8265, 8440, 8480-8481, 8490, 8510, 8560-8562 and 8570-8576). Non-adenocarcinomas and cases diagnosed on autopsy or death certificates were excluded.
